# Supplementary material for: Association between national action and trends in antibiotic resistance: an analysis of 73 countries from 2000 to 2023
Source: PLOS Glob Public Health. 2025 Apr 30;5(4):e0004127. doi: 10.1371/journal.pgph.0004127 (PMC12043137; doi:10.1371/journal.pgph.0004127)
Supplement: S1 Text — (PDF) [file pgph.0004127.s001.pdf]

S1 Text. Supplementary Methods

All code and data necessary for the analysis is available at https://github.com/PSJorgensen/national-actions-and-trends-in-antibiotic-resistance. The results can also be viewed in an interactive web app at https://gedb.shinyapps.io/amr_trend/.

Description of DPSEA indicators.

Drivers – health system: Driving forces behind human antibiotic use is captured by analyzing the trends in time series data for fifteen variables across four tier 2 indicators including infection prevalence (primary driver), sanitation standards, vaccination coverage, and health care workforce. Total data for drivers are compiled for a total of 219 countries from the United Nations (UN), World Bank database and the World Health Organization (WHO). Data availability varies by indicator as detailed in S1 Table.

Pressure - antibiotic use (ABU): Data from IQVIA database [1] are estimates of the total volume of sales of each antibiotic molecule (or combination of molecules) based on national sample surveys of antibiotic sales. Antibiotic consumption data are in kilograms and converted into defined daily doses (DDDs) using the Anatomical Therapeutic Chemical Classification System (ATC/DDD, 2016) developed by the WHO Collaborating Centre for Drug Statistics Methodology as in Klein et al. [2]. Newly Available Antibiotic Use defined as antibiotics first introduced in 1999 or later [2].

State - resistance: Data obtained from ResistanceMap [3] which is a repository of global antimicrobial resistance data. ResistanceMap obtains data from public and private sources, including lab networks, hospitals, and government agencies. Data include resistance rates for eight high-priority pathogens isolated from blood and cerebrospinal fluid of patients and are aggregated at the country level on an annual basis. Data on ResistanceMap has been harmonized to present similar definitions of resistance across countries and regions to enable comparisons between countries.

Exposure – Drug resistance index: The Drug Resistance Index (DRI) combines use and resistance rates into a single value that provides measures of antibiotic effectiveness relative to their use [4]. While DRI has been critiqued when used as a single indicator of antibiotic effectiveness [5], we here use it as part of a multi-indicator framework. We calculated the adaptive Drug Resistance Index for countries for which data on resistance and use is available over the time

period following the methodology outlined in [4] and [6]. Briefly, the annual DRI was estimated for each country by the following equation:

$$DRI = \sum_k \rho_k^{i,t} q_k^{i,t} \tag{1}$$

where, for country *i* at time *t*, $\rho_k^{i,t}$ is the proportion of resistance among all included organisms to drug *k* and $q_k^{i,t}$ is the proportion of drug *k* used for their treatment in all drugs included in the index. Pathogens included in the analysis were *E. coli*, *K. pneumoniae*, *P. aeruginosa*, *S. aureus*, *E. faecium,* and *E. faecalis*. Antibiotics included in the analysis were aminoglycosides, broad-spectrum penicillin, carbapenems, cephalosporins, narrow-spectrum penicillin, and quinolones. Because not all countries had data for all combinations, we included a country if they had at least four of the six organisms, and 10 of the 17 total combinations possible (S3 Table).

Action – TrACSS: All action indicators are self-reported data from the Global Database for Tracking Antimicrobial Resistance Country Self-Assessment Survey (TrACSS) spanning the period of 2016-2023 (https://amrcountryprogress.org/). The survey responses are publicly available with the yearly updated version providing information about countries ongoing actions to live up to the global action plan on antimicrobial resistance. All answers are given on an ordinal scale from A to E (0-4).

References.

1. OneHealthTrust. ResistanceMap: Antibiotic Use. 2024 [cited 13 Sep 2024]. Available: https://resistancemap.onehealthtrust.org/AntibioticUse.php

2. Klein EY, Milkowska-Shibata M, Tseng KK, Sharland M, Gandra S, Pulcini C, et al. Assessment of WHO antibiotic consumption and access targets in 76 countries, 2000–15: an analysis of pharmaceutical sales data. The Lancet Infectious Diseases. 2021;21: 107–115. doi:10.1016/S1473-3099(20)30332-7

3. OneHealthTrust.ResistanceMap: Antibiotic resistance. 2024 [cited 13 Sep 2024]. Available: https://resistancemap.onehealthtrust.org/AntibioticResistance.php

4. Laxminarayan R, Klugman KP. Communicating trends in resistance using a drug resistance index. BMJ Open. 2011;1: e000135–e000135. doi:10.1136/bmjopen-2011-000135

5. Vandenbroucke-Grauls CMJE, Kahlmeter G, Kluytmans J, Kluytmans-Van Den Bergh M, Monnet DL, Simonsen GS, et al. The proposed Drug Resistance Index (DRI) is not a good measure of antibiotic effectiveness in relation to drug resistance. BMJ Global Health. 2019;4: 1–3. doi:10.1136/bmjgh-2019-001838

6. Pant S, Klein E, Gandra S, Laxminarayan R. Tracking Antibiotic Effectiveness Worldwide 1999–2014 Using the Drug Resistance Index. Open Forum Infectious Diseases. 2016;3: 1481. doi:10.1093/ofid/ofw172.1183
