# Supplementary material for: Association between national action and trends in antibiotic resistance: an analysis of 73 countries from 2000 to 2023
Source: PLOS Glob Public Health. 2025 Apr 30;5(4):e0004127. doi: 10.1371/journal.pgph.0004127 (PMC12043137; doi:10.1371/journal.pgph.0004127)

S1 Fig. Importance Scores of Variables in Model Selection from Averaged Models. For model name descriptions and formulas see S10 and S11 Table. Interaction terms shown with ":", variables excluded from the explanatory variables shown as NA. It includes all countries regardless of income.

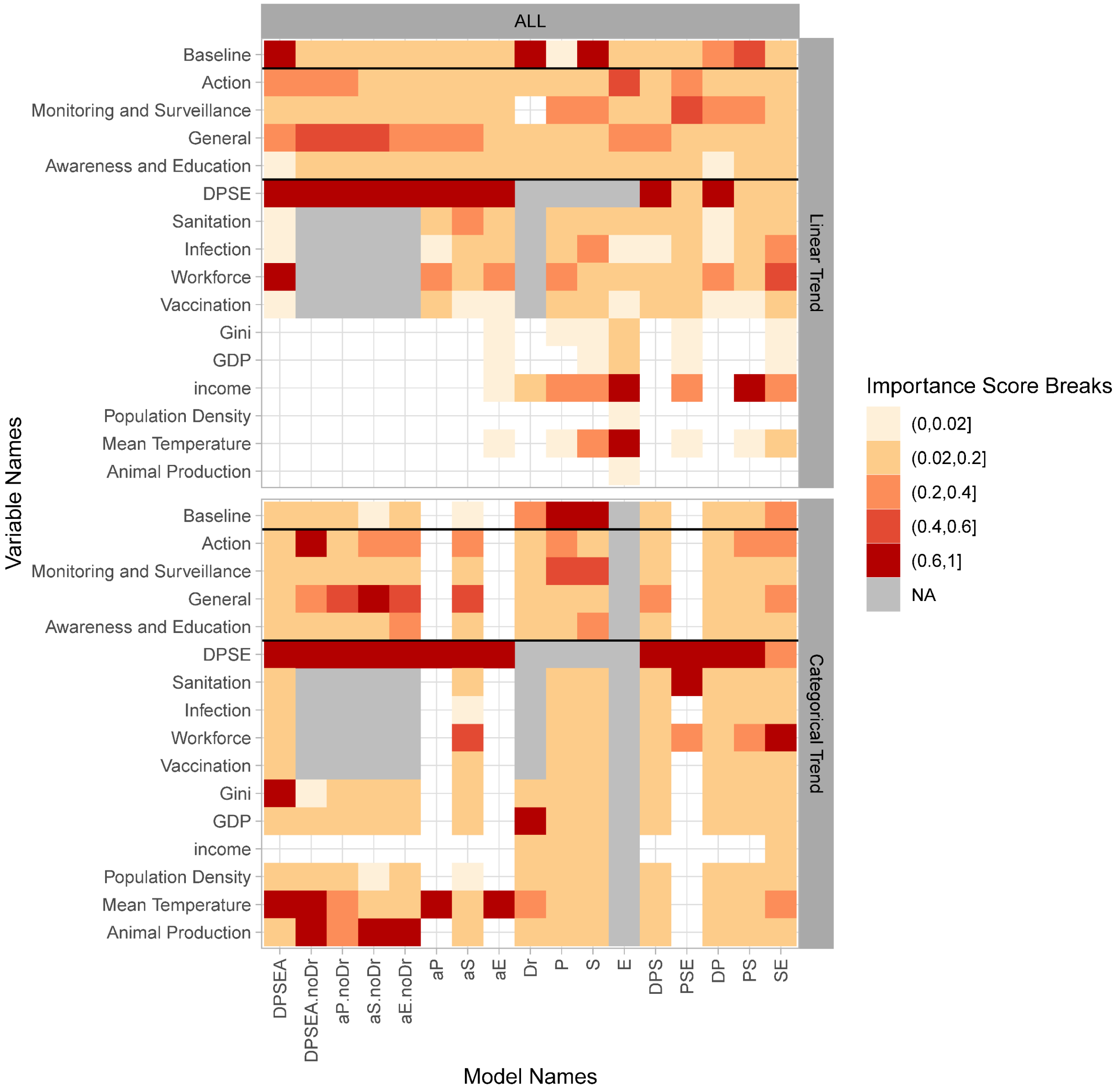

Supplement: S1 Fig — (PDF) [file pgph.0004127.s002.pdf]
