# Supplementary material for: Association between national action and trends in antibiotic resistance: an analysis of 73 countries from 2000 to 2023
Source: PLOS Glob Public Health. 2025 Apr 30;5(4):e0004127. doi: 10.1371/journal.pgph.0004127 (PMC12043137; doi:10.1371/journal.pgph.0004127)

S3 Fig. Coefficient Estimates of Variables from Best Selected Models from Model Selection. For model names see and formulas see S10 and S11 Table. Figure only included main variables. Variables excluded from the explanatory variables shown as NA. It includes all countries regardless of income.

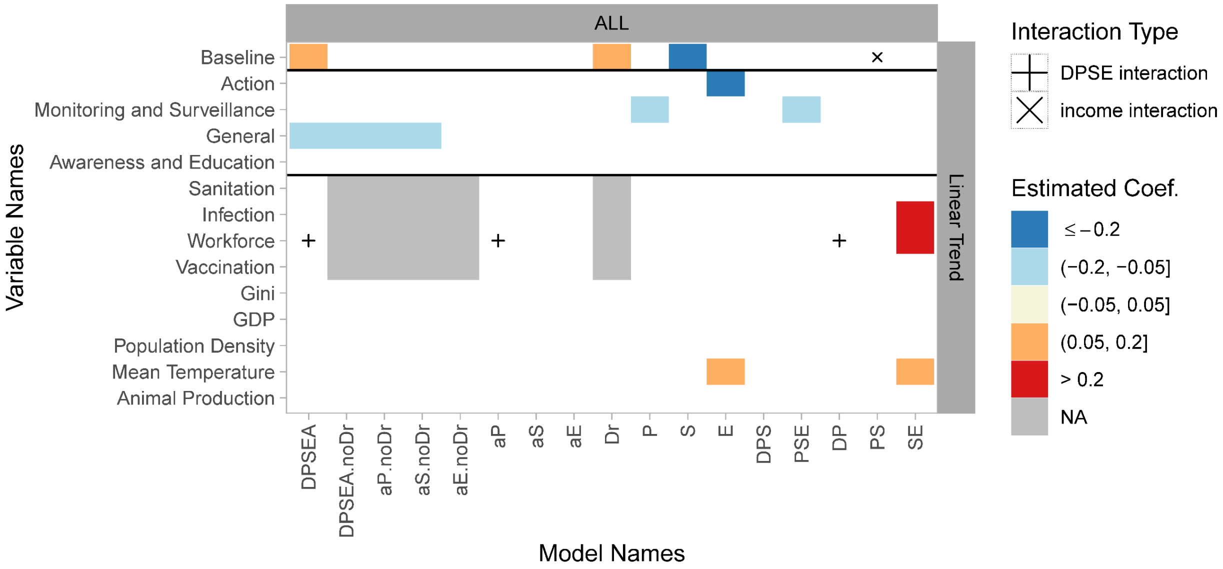

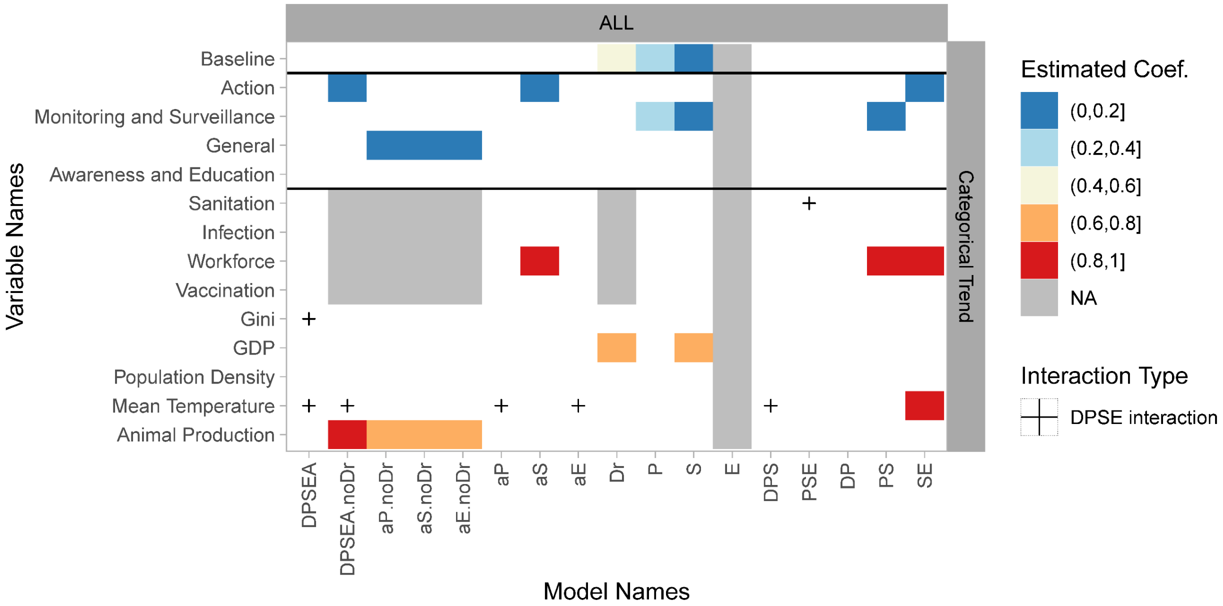

Supplement: S3 Fig — (PDF) [file pgph.0004127.s004.pdf]
