# Supplementary material for: Association between national action and trends in antibiotic resistance: an analysis of 73 countries from 2000 to 2023
Source: PLOS Glob Public Health. 2025 Apr 30;5(4):e0004127. doi: 10.1371/journal.pgph.0004127 (PMC12043137; doi:10.1371/journal.pgph.0004127)

S4 Fig. Rank of Variables in Averaged Models for Different Income

For model name descriptions and formulas see S10 and S11 Table. Interaction terms shown with ":". Variables excluded from the explanatory variables shown as NA. HIC refers to High Income Countries, LMIC refers to Low-and-Middle Income Countries.

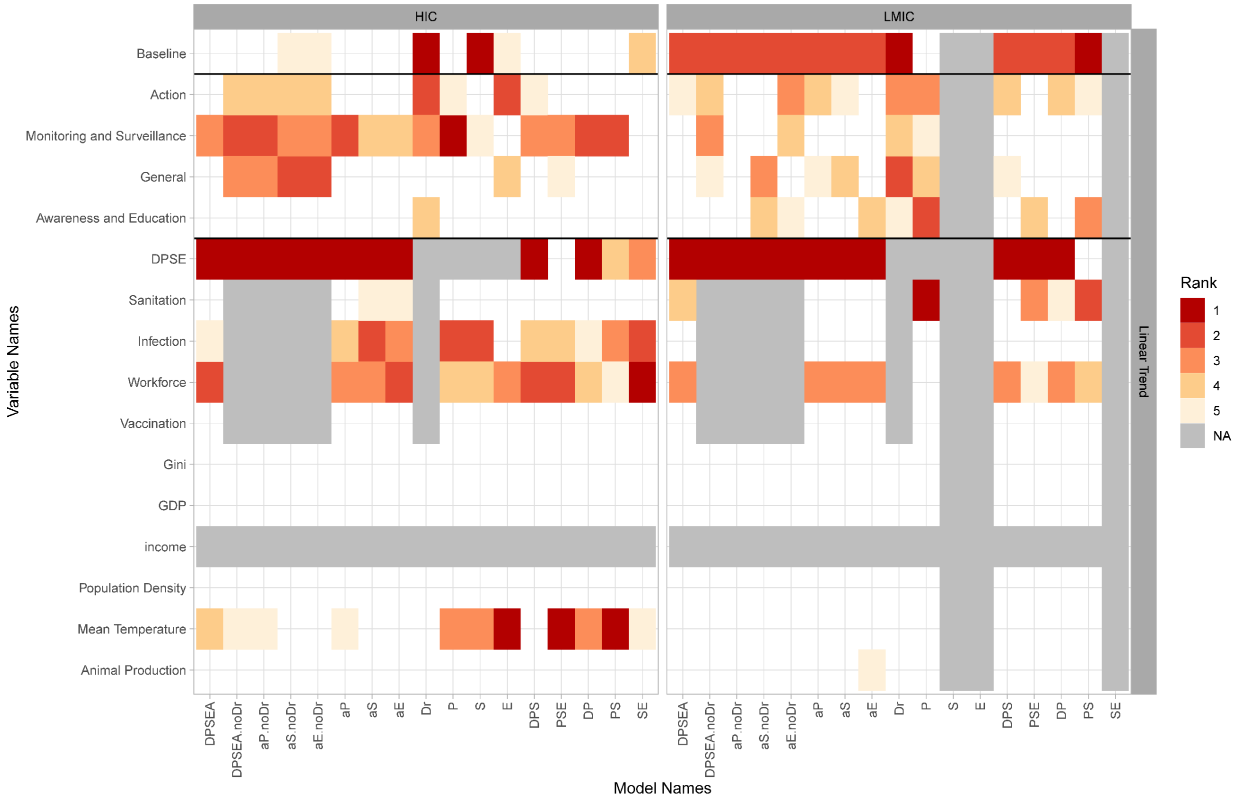

Supplement: S4 Fig — (PDF) [file pgph.0004127.s005.pdf]
