# Supplementary material for: Association between national action and trends in antibiotic resistance: an analysis of 73 countries from 2000 to 2023
Source: PLOS Glob Public Health. 2025 Apr 30;5(4):e0004127. doi: 10.1371/journal.pgph.0004127 (PMC12043137; doi:10.1371/journal.pgph.0004127)

S6 Fig. Coefficient Estimates of Averaged Models for Countries with Different Income Groups

For model name descriptions and formulas see S10 and S11 Table. Variables excluded from the explanatory variables shown as NA. HIC refers to High Income Countries, LMIC refers to Low-and-Middle Income Countries.

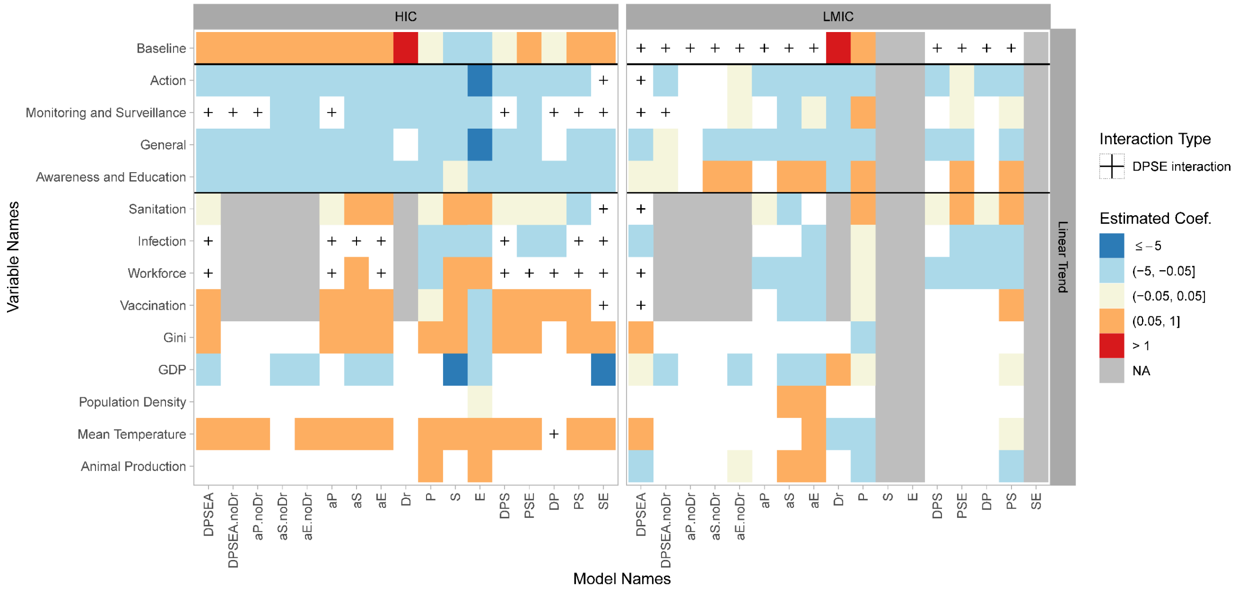

Supplement: S6 Fig — (PDF) [file pgph.0004127.s007.pdf]
