# Supplementary material for: Association between national action and trends in antibiotic resistance: an analysis of 73 countries from 2000 to 2023
Source: PLOS Glob Public Health. 2025 Apr 30;5(4):e0004127. doi: 10.1371/journal.pgph.0004127 (PMC12043137; doi:10.1371/journal.pgph.0004127)
Supplement: S1 Table — (PDF) [file pgph.0004127.s008.pdf]

S1 Table. Indicator selection for Driver categories.
Driver (as the first DPSE: Driver, Pressure, State, and Exposure indicator) data was available for 148 countries. However, countries which had driver data only included if they had one of the other indicator categories (Use, Resistance, and DRI), hence only 73 countries included in drivers (data is standardised SD=1 by denoting the ratio of standard deviation to mean). Driver data included 3 tiers. Average of tier 3 indicators are taken from different data sources.

TIER 1	TIER 2	TIER 3	DESCRIPTION	SOURCE	UNIT
**Drivers**	**Sanitation: Hygiene and Sanitation Standards (+)**	Drinking Water Source	Proportion of population using improved drinking water sources	United Nations	pct (%)
		Drinking Water Source	Population using improved drinking-water sources (%)	United Nations	pct (%)
		Overall Sanitation	Population using improved sanitation facilities (%)	United Nations	pct (%)
		Water Source Access	Improved water source (% of population with access)	United Nations	pct (%)
	**Infection: Infection Prevalence (-)**	HIV Prevalence	UN Prevalence of HIV Total (% of population ages 15-49)	United Nations	pct (%)
		HIV Prevalence	WB Prevalence of HIV Total (% of population ages 15-49)	World Bank	pct (%)
		Tuberculosis Prevalence (Tub)	UN Prevalence of tuberculosis (per 100 000 population	United Nations	per 100 000
		Incidence of Tuberculosis (Tub)	WB Incidence of tuberculosis (per 100,000 people)	World Bank	per 100 000
	**Vaccination**: **Vaccination Coverage (+)**	DTP3	Diphtheria, Tetanus, and Pertussis) immunization coverage among 1-year-olds (%)	World Health Organization	pct (%)
		HepB3	Hepatitis B) immunization coverage among 1-year-olds (%)	World Health Organization	pct (%)

		Hib3	Haemophilus influenzae type b) immunization coverage among 1-year-olds (%)	World Health Organization	pct (%)
		PCV3	Pneumococcal conjugate vaccines immunization coverage among 1-year-olds (%)	World Health Organization	pct (%)
		Pol3	Polio immunization coverage among 1-year-olds (%)	World Health Organization	pct (%)
		Measles	Immunization, measles (% of children ages 12-23 months)	United Nations and World Bank data average	pct (%)
		RCV1	Rubella vaccination coverage	World Health Organization	pct (%)
	**Workforce: Health Care Workforce** (+)	Physicians per capita	Physicians density (per 1000 population)	United Nations	per 1000
		Physicians per capita	Physicians density (per 1000 population)	World Health Organization	per 1000
		Nursing and midwifery	Nursing and midwifery personnel density per 1000 population	World Health Organization	per 1000
