# Supplementary material for: Association between national action and trends in antibiotic resistance: an analysis of 73 countries from 2000 to 2023
Source: PLOS Glob Public Health. 2025 Apr 30;5(4):e0004127. doi: 10.1371/journal.pgph.0004127 (PMC12043137; doi:10.1371/journal.pgph.0004127)
Supplement: S2 Table — (PDF) [file pgph.0004127.s009.pdf]

S2 Table. Indicator selection for Use and Resistance categories.
Use or resistance data were available for 73 countries with antibiotic usage (ABU) and antibiotic resistance (ABR) available for 65 and 32 countries, respectively. Data is standardised to the ratio of standard deviation (SD=1). For NewABXUse, year indicates year of introduction of antibiotic.

TIER 1	TIER 2	ANTIBIOTIC OR BACTERIA	SOURCE	UNIT
**Antibiotic use (ABU)**	BroadPerTotalABXUse: Use of broad-spectrum antibiotics	Fluoroquinolones, Macrolides, third-generation cephalosporins, co-amoxiclav, clindamycin, oral vancomycin	IQVIA	Percentage of total use
	NewABXUse: Newly available antibiotics	dalfopristin/ quinupristin (2005), gatifloxacin (1999), moxifloxacin (1999), linezolid (2000), telithromycin (2001), balofloxacin (2006), Biapenem (2002), ertapenem (2002), pazufloxacin (2002), prulifloxacin (2002), daptomycin (2003), gemifloxacin (2003), doripenem (2005), tigecycline (2005), garenoxacin (2007), ceftobiprole (2008), sitafloxacin (2008), tebipenem (2009), telavancin (2009), antofloxacin (2010), ceftaroline (2011), ceftolozane/tazobactam (2014), dalbavancin (2014), oritavancin (2014), tedizolid (2014)	IQVIA	Defined Daily Doses (DDD)
	Total use: Total Antibiotic Use Per Capita	-	IQVIA	DDD per 1000 capita
**Antibiotic resistance (ABR)**	MRSA: Methicillin-resistant Staphylococcus aureus	-	ResistanceMap	percent (%)
	CR: Carbapenem resistance (average)	Enterobacteriaceae and other bacteria	ResistanceMap	percent (%)
	STR: Streptococcal resistance (average)	Macrolides and penicillin	ResistanceMap	percent (%)
