# Supplementary material for: Association between national action and trends in antibiotic resistance: an analysis of 73 countries from 2000 to 2023
Source: PLOS Glob Public Health. 2025 Apr 30;5(4):e0004127. doi: 10.1371/journal.pgph.0004127 (PMC12043137; doi:10.1371/journal.pgph.0004127)
Supplement: S3 Table — (PDF) [file pgph.0004127.s010.pdf]

S3 Table. Indicator selection for DRI (exposure) category.

The drug resistance index (DRI) was calculated for 25 countries for the pathogen and antibiotics combinations listed below. Data is standardized by the standard deviation (SD=1).

PATHOGEN	ANTIBIOTICS	SOURCE	UNIT
Enterococcus faecalis/faecium	Aminopenicillins	IQVIA, ResistanceMap	Unit Free
Escherichia coli	Aminoglycosides<br>Aminopenicillins<br>Carbapenems<br>Cephalosporins (3rd gen)<br>Fluoroquinolones	IQVIA, ResistanceMap	Unit Free
Klebsiella pneumonia	Aminoglycosides<br>Carbapenems<br>Cephalosporins (3rd gen)<br>Fluoroquinolones	IQVIA, ResistanceMap	Unit Free
Pseudomonas aeruginosa	Aminoglycosides<br>Carbapenems<br>Cephalosporins (3rd gen)<br>Fluoroquinolones<br>Piperacillin-tazobactam	IQVIA, ResistanceMap	Unit Free
Staphylococcus aureus	Oxacillin/Cefoxitin (MRSA)	IQVIA, ResistanceMap	Unit Free
