# Supplementary material for: Association between national action and trends in antibiotic resistance: an analysis of 73 countries from 2000 to 2023
Source: PLOS Glob Public Health. 2025 Apr 30;5(4):e0004127. doi: 10.1371/journal.pgph.0004127 (PMC12043137; doi:10.1371/journal.pgph.0004127)
Supplement: S4 Table — (PDF) [file pgph.0004127.s011.pdf]

S4 Table. Ecological variables used as covariates.

Ecological variables are the are non-related variables to DPSE indicators and referred as covariates in the analyses. Covariates are grouped according to their context.

CONTEXT	COVARIATES	DESCRIPTION	SOURCE	UNIT
**Economy**	Gross Domestic Product	Purchasing power parity, 2015 base year.	The Eora Global Supply Chain Database	Log10 US dollars per capita
**Economy**	Gini	Gini index: Inequality – proportion of the lowest 20 % of the national income distribution (2004-2012)	World Income Inequality Database - WIID	Unit free (0:1)
**Climate**	Mean Temperature	Mean temperature weighted by population density of 1x1 degree grid cells	NASA's Socioeconomic Data and Applications Center (SEDAC), NASA Center for Climate Simulation (BioClim)	degree C
**Livestock**	Animal Production	Animal production in mass per country area	Gridded Livestock of the World (GLW)	tonnes per km^2
**Population**	Population Density	Human population density	NASA's Socioeconomic Data and Applications Center (SEDAC)	Log10 (population/km^2)
