# Supplementary material for: Association between national action and trends in antibiotic resistance: an analysis of 73 countries from 2000 to 2023
Source: PLOS Glob Public Health. 2025 Apr 30;5(4):e0004127. doi: 10.1371/journal.pgph.0004127 (PMC12043137; doi:10.1371/journal.pgph.0004127)
Supplement: S5 Table — (PDF) [file pgph.0004127.s012.pdf]

S5 Table. List of countries included in the study.

The total number of countries included in the study is 73. ISO3 codes refers to three-letter country codes according to ISO 3166-1. Income levels: HIC refers for High Income Countries, LMIC refers for Low- and Middle-Income Countries.

ISO3	Country	Income
**Asia**		
ARE	United Arab Emirates	HIC
BGD	Bangladesh	LMIC
CHN	China	LMIC
IDN	Indonesia	LMIC
IND	India	LMIC
JOR	Jordan	LMIC
JPN	Japan	HIC
KAZ	Kazakhstan	LMIC
KOR	Republic of Korea	HIC
LBN	Lebanon	LMIC
LKA	Sri Lanka	LMIC
MYS	Malaysia	LMIC
PAK	Pakistan	LMIC
PHL	Philippines	LMIC
SAU	Saudi Arabia	HIC
SGP	Singapore	HIC
THA	Thailand	LMIC
TUR	Turkey	LMIC
UKR	Ukraine	LMIC
VNM	Vietnam	LMIC
**South America**		
ARG	Argentina	LMIC
BRA	Brazil	LMIC
CHL	Chile	LMIC
COL	Colombia	LMIC
ECU	Ecuador	LMIC
PER	Peru	LMIC
URY	Uruguay	LMIC
**Oceania**		
AUS	Australia	HIC
NZL	New Zealand	HIC
**Europe**		
AUT	Austria	HIC
BEL	Belgium	HIC
BGR	Bulgaria	LMIC
BLR	Belarus	LMIC

ISO3	Country	Income
CHE	Switzerland	HIC
CYP	Cyprus	HIC
CZE	Czech Republic	HIC
DEU	Germany	HIC
DNK	Denmark	HIC
ESP	Spain	HIC
EST	Estonia	HIC
FIN	Finland	HIC
FRA	France	HIC
GBR	United Kingdom	HIC
GRC	Greece	HIC
HRV	Croatia	HIC
HUN	Hungary	HIC
IRL	Ireland	HIC
ISL	Iceland	HIC
ITA	Italy	HIC
LTU	Lithuania	LMIC
LUX	Luxembourg	HIC
LVA	Latvia	HIC
MKD	North Macedonia	LMIC
MLT	Malta	HIC
NLD	Netherlands	HIC
NOR	Norway	HIC
POL	Poland	HIC
PRT	Portugal	HIC
ROU	Romania	LMIC
RUS	Russian Federation	LMIC
SRB	Serbia	LMIC
SVK	Slovakia	HIC
SVN	Slovenia	HIC
SWE	Sweden	HIC
**North America**		
CAN	Canada	HIC
DOM	Dominican Republic	LMIC
MEX	Mexico	LMIC
USA	United States of America	HIC
**Africa**		
EGY	Egypt	LMIC
GHA	Ghana	LMIC
MAR	Morocco	LMIC
TUN	Tunisia	LMIC
ZAF	South Africa	LMIC
