# Supplementary material for: Association between national action and trends in antibiotic resistance: an analysis of 73 countries from 2000 to 2023
Source: PLOS Glob Public Health. 2025 Apr 30;5(4):e0004127. doi: 10.1371/journal.pgph.0004127 (PMC12043137; doi:10.1371/journal.pgph.0004127)
Supplement: S6 Table — (PDF) [file pgph.0004127.s013.pdf]

S6 Table. Governance Syndrome questions.

Questions used and their sectors included for the calculation of governance syndrome for the years between 2016-2022 according to TrACSS (1.1) 2016-2017 version. All surveys are available online (https://amrcountryprogress.org/#/download-responses). Only questions asked in all surveys were selected for the analysis. In case a question is separated in two questions, they were still kept for the analysis. Answers converted from the scale of A-E to 0-4. Categories' scores were averaged from their constituted questions, and the overall governance score was calculated as the mean score of all categories. NAP stands for National Action Plan.

ACTION SUB-CATEGORIES		AMR governance response survey						
**Category**	**Question title**	2016–2017	2017–2018	2018–2019	2019–2020	2020–2021	2021–2022	2022–2023
**General**	Multi-sector and One Health working arrangements	4.1	4.1	4.1	4.1	4.1	2.1	2.1
	Country progress with development of a national action plan on AMR	5.1	5.1	5.1	5.1	5.1	2.3	2.3
**Awareness and Education**	Raising awareness and understanding of AMR risks and response	6.1, 6.2	6.1, 6.2	6.1	6.1	6.1	2.9	2.9
	Training and professional education on AMR in the human health sector	6.3	6.3	6.2	6.2	6.2	3.1	3.1
	Training and professional education on AMR in the veterinary sector	6.4	6.4	6.3	6.3	6.3	4.1	4.1
	Progress with strengthening veterinary services	6.5	6.5	6.5	6.5	6.5	4.3	4.3
**Monitoring and Surveillance**	National monitoring system for consumption and rational use of antimicrobials in human health	7.1	7.1	7.1	7.1	7.1	3.2	3.2
	National surveillance system for antimicrobial resistance (AMR) in humans	7.3	7.4	7.4	7.4	7.4	3.3	3.3
**Prevention**	Infection Prevention and Control (IPC) in human health care	8.1	8.1	8.1	8.1	8.1	3.5	3.5
	Good health, management and hygiene practices to reduce the use of antimicrobials and minimize development and transmission of AMR in animal production (terrestrial and aquatic)	8.2	8.2	8.2	8.2	8.2	4.9, 4.10	4.9, 4.10
**Regulation**	Antimicrobial Stewardship & regulation in human health	9.1	9.1	9.1	9.1	9.1	3.6	3.6
	Antimicrobial stewardship & regulation in terrestrial animal health	9.2	9.2	9.2	9.2	9.2	4.11	4.11
