# Supplementary material for: Association between national action and trends in antibiotic resistance: an analysis of 73 countries from 2000 to 2023
Source: PLOS Glob Public Health. 2025 Apr 30;5(4):e0004127. doi: 10.1371/journal.pgph.0004127 (PMC12043137; doi:10.1371/journal.pgph.0004127)
Supplement: S7 Table — (PDF) [file pgph.0004127.s014.pdf]

**S7 Table. Questions used for calculating the action index.**

Topics and titles of questions from the TrACSS survey in 2016-17 used to calculate the action index as well as the sub-categories used to group questions within a similar theme. Answers were answered on a scale of A-E and converted to 0-4.

| Topic | Survey Questions |
|---|---|
| **Awareness and Education** | |
| AMR Training (Human Health) * | 6.3 Training and professional education on AMR in the human health sector |
| AMR Training (Animal Health and Food Production) * | 6.4 Training and professional education on AMR in the veterinary sector |
| AMR Awareness (Human Health) * | 6.1 Raising awareness and understanding of AMR risks and response in human health |
| AMR Awareness (Animal Health and Food Production) * | 6.2 Raising Awareness and understanding of AMR risks and response in animal health and food production. |
| **Monitoring and Surveillance** | |
| Monitoring System for AMU (Animals and Crop) * | 7.2 National monitoring system for antimicrobial use in animals and crop production |
| AMR Surveillance System (Humans)* | 7.3 National surveillance system for antimicrobial resistance (AMR) in humans |
| Monitoring System for AMU (Human Health) * | 7.1 National monitoring system for consumption and rational use of antimicrobials in human health |
| AMR Surveillance System (Animals and Foods) * | 7.4 National surveillance system for antimicrobial resistance (AMR) in animals and foods |
| **General** | |
| Veterinary Services | 6.5 Progress with strengthening veterinary services |
| One Health Arrangements | 4.1 Multi-sector and One Health working arrangement |
| NAP progress | 5.1 Country progress with development of a national action plan on AMR |
| **Regulation** | |
| AMS and Regulation (Human)* | 9.1 Antimicrobial Stewardship & regulation in human health |
| AMS and Regulation (Animal & Crop) * | 9.2 Antimicrobial stewardship & regulation in animal and crop production |
| Contamination Prevention | 9.3 Legislation and/or regulations to prevent contamination of the environment with antimicrobials |
| **Prevention** | |
| IPC | 8.1 Infection Prevention and Control (IPC) in human health care |
| AMU Prevention | 8.2 Good animal health and management practices and good hygiene to prevent infections in order to reduce the use of antimicrobials in animals and AMR transmission in food production |
| **Subcategories included in Animal Data Analysis and Figure 4* | |
