# Supplementary material for: Association between national action and trends in antibiotic resistance: an analysis of 73 countries from 2000 to 2023
Source: PLOS Glob Public Health. 2025 Apr 30;5(4):e0004127. doi: 10.1371/journal.pgph.0004127 (PMC12043137; doi:10.1371/journal.pgph.0004127)
Supplement: S8 Table — (PDF) [file pgph.0004127.s015.pdf]

S8 Table. Model Formulas for Association between Action and Indicator Linear Trend and Categorical Trend.

Formulas for generalized linear mixed models to investigate the association between linear trend of indicators in 16 years and action. First type of model included linear trend of indicators as response variable. The second type of model included Action as a response variable and categorical trend as an explanatory variable to investigate the action difference between the countries with positive vs negative change. Results shown in Figure 2 for tier 1 indicators.

**Indicators**	**Formula**
**Drivers**	Linear trend ~ Action + Baseline + (1|Income)
	Action ~ Categorical trend + Baseline + (1|Income)
**Use**	Linear trend ~ Action + Baseline + (1|Income)
	Action ~ Categorical trend + Baseline + (1|Income)
**Resistance**	Linear trend ~ Action + Baseline + (1|Income)
	Action ~ Categorical trend + Baseline + (1|Income)
**DRI**	Linear trend ~ Action + Baseline + (1|Income)
	Action ~ Categorical trend + Baseline + (1|Income)
