# Supplementary material for: Association between national action and trends in antibiotic resistance: an analysis of 73 countries from 2000 to 2023
Source: PLOS Glob Public Health. 2025 Apr 30;5(4):e0004127. doi: 10.1371/journal.pgph.0004127 (PMC12043137; doi:10.1371/journal.pgph.0004127)
Supplement: S9 Table — (PDF) [file pgph.0004127.s016.pdf]

S9 Table. De-escalation plot formulas for univariate models.

Formulas used for binomial general linear model to investigate the association between de-escalation of categories and action level. Proportion in decline refers to declining numbers of tier 2 indicators divided by the total number of tier 2 indicators for countries, Baseline Mean refers to mean of baseline for tier 2 indicators for each DPSE indicator (See S1-S3 Table). Models weighted by total number of tier 2 indicators within each DPSE indicator for specific country.

**Indicators**	**Formula**	**Weight**
**Drivers**	Proportion in decline ~ Action+ Baseline Mean	(1-4)/4
**Use**	Proportion in decline ~ Action+ Baseline Mean	(1-3)/3
**Resistance**	Proportion in decline ~ Action+ Baseline Mean	(1-3)/3
**DRI**	Proportion in decline ~ Action+ Baseline Mean	1/1
