# Supplementary material for: Association between national action and trends in antibiotic resistance: an analysis of 73 countries from 2000 to 2023
Source: PLOS Glob Public Health. 2025 Apr 30;5(4):e0004127. doi: 10.1371/journal.pgph.0004127 (PMC12043137; doi:10.1371/journal.pgph.0004127)
Supplement: S10 Table — (PDF) [file pgph.0004127.s017.pdf]

S10 Table. Global Models Data Subset Formulas for The Model Selection
Global Models for multivariate model selection data subsets.

**Model Name**	**Description**
DPSEA	All DPSE indicators as response variables.
DPSEA.noDr	All DPSE indicators as response variables, explanatory variables relating to health system excluded.
aP.noDr	Only countries reporting use (P) included, all DPSE indicators as response variable, explanatory variables relating to health system excluded.
aS.noDr	Only countries reporting resistance (S) included, all DPSE indicators as response variable, explanatory variables relating to health system excluded.
aE.noDr	Only countries reporting DRI (E) included, all DPSE indicators as response variable, explanatory variables relating to health system excluded.
aP	Only countries reporting use (P) included, all DPSE indicators as response variable.
aS	Only countries reporting resistance (S) included, all DPSE indicators as response variable.
aE	Only countries reporting DRI (E), all DPSE indicators as response variable.
Dr	Only countries with tier 2 driver components and at least one of Use (P), Resistance (S) or DRI (E) included. Only driver indicators as response variable.
P	Only countries with tier 2 use (P) components included. Only use indicators as response variable.
S	Only countries with tier 2 resistance (S) components included. Only resistance indicators as response variable.
E	Only countries with DRI (E) included. Only DRI indicators as response variable.
DPS	Only countries with reporting driver, use (P), or resistance (S) included. Driver, use, and resistance as a response variable included.
PSE	Only countries with reporting use (P), resistance (S) or DRI (E) included. use, resistance, and DRI as a response variable included.
DP	Only countries with reporting driver or use (P) included. Driver and use as a response variable included.
PS	Only countries with reporting, use (P), or resistance (S) included. Use and resistance as a response variable included.
SE	Only countries with reporting resistance (S) or DRI (E) included. Resistance and DRI as a response variable included.
