# Supplementary material for: Association between national action and trends in antibiotic resistance: an analysis of 73 countries from 2000 to 2023
Source: PLOS Glob Public Health. 2025 Apr 30;5(4):e0004127. doi: 10.1371/journal.pgph.0004127 (PMC12043137; doi:10.1371/journal.pgph.0004127)
Supplement: S11 Table — (PDF) [file pgph.0004127.s018.pdf]

S11 Table. Model selection global model formulas

Global models as starting points for creating and evaluating model subsets.

Model Name	Global Models with DPSE interaction	Global Models with income interaction
DPSEA<br>aP<br>aS<br>aE<br>DPS<br>PSE<br>DP<br>PS<br>SE	Linear trend ~ Action * DPSE + General * DPSE + Monitoring and Surveillance * DPSE + Awareness and Education * DPSE + Sanitation * DPSE + Infection * DPSE + Workforce * DPSE + Vaccination * DPSE + GDP * DPSE + Pop. density * DPSE + Gini * DPSE + Animal prod. * DPSE + Mean temperature * DPSE + Baseline * DPSE + (1 | ISO3)	Linear trend ~ Action * income + General * income + Monitoring and Surveillance * income + Awareness and Education * income + Sanitation * income + Infection * income + Workforce * income + Vaccination * income + GDP * income + Pop. density * income + Gini * income + Animal prod. * income + Mean temperature * income + Baseline * income + (1 | ISO3)
DPSEA.noDr<br>aP.noDr<br>aS.noDr<br>aE.noDr	Linear trend ~ Action * DPSE + General * DPSE + Monitoring and Surveillance * DPSE + Awareness and Education * DPSE + GDP * DPSE + Pop. density * DPSE + Gini * DPSE + Animal prod. * DPSE + Mean temperature * DPSE + Baseline * DPSE + (1 | ISO3)	Linear trend ~ Action * income + General * income + Monitoring and Surveillance * income + Awareness and Education * income + GDP * income + Pop. density * income + Gini * income + Animal prod. * income + Mean temperature * income + Baseline * income + (1 | ISO3)
Dr	No model	Linear trend ~ Action*income + General*income + Monitoring and Surveillance*income + Awareness and Education*income + GDP*income + Pop. density*income + Gini*income + Animal prod.*income + Mean temperature*income + Baseline*income + (1|ISO3) + (1|Subcategory)
P<br>S	No model	Linear trend ~ Action*income + General*income + Monitoring and Surveillance*income + Awareness and Education*income + Sanitation * income + Infection * income + Workforce * income + Vaccination * income + GDP*income + Pop. density*income + Gini*income + Animal prod.*income + Mean temperature*income + Baseline*income + (1|ISO3) + (1|Subcategory)
E	No model	Linear trend ~ Action * income + General * income + Monitoring and Surveillance * income + Awareness and Education * income + Sanitation * income + Infection * income + Workforce * income + Vaccination * income + GDP * income + Pop. density * income + Gini * income + Animal prod. * income + Mean temperature * income + Baseline * income
**HIC**		
DPSEA<br>aP<br>aS<br>aE<br>DPS<br>PSE<br>DP<br>PS<br>SE	Linear trend ~ Action * DPSE + General * DPSE + Monitoring and Surveillance * DPSE + Awareness and Education * DPSE + Sanitation * DPSE + Infection * DPSE + Workforce * DPSE + Vaccination * DPSE + GDP * DPSE + Pop. density * DPSE + Gini * DPSE + Animal prod. * DPSE + Mean temperature * DPSE + Baseline * DPSE + (1 | ISO3)	No model

Model Name	Global Models with DPSE interaction	Global Models with income interaction
DPSEA.noDr<br>aP.noDr<br>aS.noDr<br>aE.noDr	Linear trend ~ Action * DPSE + General * DPSE + Monitoring and Surveillance * DPSE + Awareness and Education * DPSE + GDP * DPSE + Pop. density * DPSE + Gini * DPSE + Animal prod. * DPSE + Mean temperature * DPSE + Baseline * DPSE + (1 | ISO3)	No model
Dr	Linear trend ~ Action + General + Monitoring and Surveillance + Awareness and Education + GDP + Pop. density + Gini + Animal prod. + Mean temperature + Baseline + (1 | ISO3) + (1 | Subcategory)	No model
P<br>S<br>E	Linear trend ~ Action + General + Monitoring and Surveillance + Awareness and Education + Sanitation + Infection + Workforce + Vaccination + GDP + Pop. density + Gini + Animal prod. + Mean temperature + Baseline + (1 | ISO3) + (1 | Subcategory)	No model
**LMIC**		No model
DPSEA<br>aP<br>aS<br>aE<br>DPS<br>PSE<br>DP<br>PS	Linear trend ~ Action * DPSE + General * DPSE + Monitoring and Surveillance * DPSE + Awareness and Education * DPSE + Sanitation * DPSE + Infection * DPSE + Workforce * DPSE + Vaccination * DPSE + GDP * DPSE + Pop. density * DPSE + Gini * DPSE + Animal prod. * DPSE + Mean temperature * DPSE + Baseline * DPSE + (1 | ISO3)	No model
DPSEA.noDr<br>aP.noDr<br>aS.noDr<br>aE.noDr	Linear trend ~ Action * DPSE + General * DPSE + Monitoring and Surveillance * DPSE + Awareness and Education * DPSE + GDP * DPSE + Pop. density * DPSE + Gini * DPSE + Animal prod. * DPSE + Mean temperature * DPSE + Baseline * DPSE + (1 | ISO3)	No model
Dr	Linear trend ~ Action + General + Monitoring and Surveillance + Awareness and Education + GDP + Pop. density + Gini + Animal prod. + Mean temperature + Baseline + (1 | ISO3) + (1 | Subcategory)	No model
P	Linear trend ~ Action + General + Monitoring and Surveillance + Awareness and Education + Sanitation + Infection + Workforce + Vaccination + GDP + Pop. density + Gini + Animal prod. + Mean temperature + Baseline + (1 | ISO3) + (1 | Subcategory)	No model
E	No model	No model

Model Name	Global Models with DPSE interaction	Global Models with income interaction
SE	No model	No model
**BINOMIAL**		
DPSEA<br>aP<br>aS<br>aE	Categorical trend ~ Action * DPSE + General * DPSE + Monitoring and Surveillance * DPSE + Awareness and Education * DPSE + Sanitation * DPSE + Infection * DPSE + Workforce * DPSE + Vaccination * DPSE + GDP * DPSE + Pop. density * DPSE + Gini * DPSE + Animal prod. * DPSE + Mean temperature * DPSE + Baseline * DPSE + (1 | ISO3)	Categorical trend ~ Action * income + General * income + Monitoring and Surveillance * income + Awareness and Education * income + Sanitation * income + Infection * income + Workforce * income + Vaccination * income + GDP * income + Pop. density * income + Gini * income + Animal prod. * income + Mean temperature * income + Baseline * income + (1 | ISO3)
DPSEA.noDr<br>aP.noDr<br>aE.noDr	Categorical trend ~ Action * DPSE + General * DPSE + Monitoring and Surveillance * DPSE + Awareness and Education * DPSE + GDP * DPSE + Pop. density * DPSE + Gini * DPSE + Animal prod. * DPSE + Mean temperature * DPSE + Baseline * DPSE + (1 | ISO3)	Categorical trend ~ Action * income + General * income + Monitoring and Surveillance * income + Awareness and Education * income + GDP * income + Pop. density * income + Gini * income + Animal prod. * income + Mean temperature * income + Baseline * income + (1 | ISO3)
aS.noDr	Categorical trend ~ Action * DPSE + General * DPSE + Monitoring and Surveillance * DPSE + Awareness and Education * DPSE + GDP * DPSE + Pop. density * DPSE + Gini * DPSE + Animal prod. * DPSE + Baseline * DPSE + (1 | ISO3); Categorical trend ~ Action * DPSE + General * DPSE + Monitoring and Surveillance * DPSE + Awareness and Education * DPSE + GDP * DPSE + Pop. density * DPSE + Gini * DPSE + Mean temperature * DPSE + Baseline * DPSE + (1 | ISO3); Categorical trend ~ Action * DPSE + General * DPSE + Monitoring and Surveillance * DPSE + Awareness and Education * DPSE + GDP * DPSE + Gini * DPSE + Animal prod. * DPSE + Mean temperature * DPSE + Baseline * DPSE + (1 | ISO3)	Categorical trend ~ Action * income + General * income + Monitoring and Surveillance * income + Awareness and Education * income + GDP * income + Pop. density * income + Gini * income + Animal prod. * income + Mean temperature * income + Baseline * income + (1 | ISO3)
Dr		Categorical trend ~ Action * income + General * income + Monitoring and Surveillance * income + Awareness and Education * income + GDP * income + Pop. density * income + Gini * income + Animal prod. * income + Mean temperature * income + Baseline * income + (1 | ISO3)
P<br>S		Categorical trend ~ Action * income + General * income + Monitoring and Surveillance * income + Awareness and Education * income + Sanitation * income + Infection * income + Workforce * income + Vaccination * income + GDP * income + Pop. density * income + Gini * income + Animal prod. * income + Mean temperature * income + Baseline * income + (1 | ISO3) + (1 | Subcategory)

Model Name	Global Models with DPSE interaction	Global Models with income interaction
E	No model	No model
DPS<br>PSE<br>DP<br>SE	Categorical trend ~ Action * DPSE + General * DPSE + Monitoring and Surveillance * DPSE + Awareness and Education * DPSE + Sanitation * DPSE + Infection * DPSE + Workforce * DPSE + Vaccination * DPSE + GDP * DPSE + Pop. density * DPSE + Gini * DPSE + Animal prod. * DPSE + Mean temperature * DPSE + Baseline * DPSE + (1 | ISO3)	Categorical trend ~ Action * income + General * income + Monitoring and Surveillance * income + Awareness and Education * income + Sanitation * income + Infection * income + Workforce * income + Vaccination * income + GDP * income + Pop. density * income + Gini * income + Animal prod. * income + Mean temperature * income + Baseline * income + (1 | ISO3)
PS	Categorical trend ~ Action * DPSE + General * DPSE + Monitoring and Surveillance * DPSE + Awareness and Education * DPSE + Sanitation * DPSE + Infection * DPSE + Workforce * DPSE + Vaccination * DPSE + GDP * DPSE + Pop. density * DPSE + Gini * DPSE + Animal prod. * DPSE + Mean temperature * DPSE + Baseline * DPSE + (1 | ISO3)	Categorical trend ~ Action * income + General * income + Monitoring and Surveillance * income + Awareness and Education * income + Sanitation * income + Infection * income + Workforce * income + Vaccination * income + Pop. density * income + Gini * income + Animal prod. * income + Mean temperature * income + Baseline * income + (1 | ISO3)**;** Categorical trend ~ General * income + Monitoring and Surveillance * income + Awareness and Education * income + Sanitation * income + Infection * income + Workforce * income + Vaccination * income + GDP * income + Pop. density * income + Gini * income + Animal prod. * income + Mean temperature * income + Baseline * income + (1 | ISO3)**;** Categorical trend ~ Action * income + Monitoring and Surveillance * income + Awareness and Education * income + Sanitation * income + Infection * income + Workforce * income + Vaccination * income + GDP * income + Pop. density * income + Gini * income + Animal prod. * income + Mean temperature * income + Baseline * income + (1 | ISO3)
