# Supplementary material for: Association between national action and trends in antibiotic resistance: an analysis of 73 countries from 2000 to 2023
Source: PLOS Glob Public Health. 2025 Apr 30;5(4):e0004127. doi: 10.1371/journal.pgph.0004127 (PMC12043137; doi:10.1371/journal.pgph.0004127)
Supplement: S12 Table — (PDF) [file pgph.0004127.s019.pdf]

S12 Table. Association Between Linear Trend and Action

Association is determined with linear mixed model between linear trend of variables as a response variable and action and baseline as fixed effects and income level as a random effect.

Indicators	DPSE	Coefficient	t-value	std.error	df	p.value	Number of Countries with Increase	Sample Size
level 1								
Drivers Total	Drivers	-0.05	-2.6	0.02	21.4	**0.017**	6	73
Use Total	Use	-0.16	-2.3	0.07	39.0	**0.024**	55	65
Resistance Total	Resistance	-0.11	-1.1	0.10	26.3	0.291	16	32
DRI	DRI	-0.17	-2.1	0.08	21.1	**0.044**	21	25
level 2								
Infections	Drivers	0.01	0.8	0.01	48.1	0.402	12	73
Sanitation	Drivers	-0.01	-0.6	0.02	66.1	0.548	27	73
Vaccination	Drivers	-0.09	-1.6	0.05	70.0	0.108	11	73
Workforce	Drivers	-0.21	-3.9	0.05	29.8	**0.001**	9	55
TotalDDDPer1000Persons	Use	0.04	0.4	0.10	61.4	0.659	50	65
BroadPerTotalABXUse	Use	-0.32	-3.3	0.10	43.7	**0.002**	47	65
NewABXUse	Use	-0.15	-1.8	0.08	60.0	0.076	55	63
MRSA	Resistance	-0.17	-1.6	0.11	29.0	0.129	11	32
CR	Resistance	-0.11	-0.6	0.20	18.7	0.587	20	28
STR	Resistance	-0.13	-1.7	0.08	22.0	0.113	13	25
level 3								
HIV	Drivers/infections	0.01	1.6	0.01	28.0	0.127	22	31
TB	Drivers/infections	0.02	1.1	0.02	27.9	0.29	11	73
Drinking Water Source	Drivers/Sanitation	0.01	0.6	0.02	69.0	0.577	65	72
Water Source Access	Drivers/Sanitation	0.01	0.6	0.02	69.0	0.533	65	72
Overall Sanitation	Drivers/Sanitation	0.01	0.8	0.02	62.4	0.435	63	66
DTP3	Drivers/Vaccination	0.11	1.8	0.06	69.0	0.081	51	72
HepB3	Drivers/Vaccination	0.13	1.5	0.09	57.0	0.148	48	60
Hib3	Drivers/Vaccination	0.02	0.3	0.08	41.9	0.796	45	53
Pol3	Drivers/Vaccination	0.13	2.3	0.06	69.0	**0.027**	49	72
Measles	Drivers/Vaccination	0.11	2.3	0.05	70.0	**0.022**	53	73
RCV1	Drivers/Vaccination	0.18	2.2	0.08	59.0	**0.033**	43	62
Nursing	Drivers/Workforce	0.19	2.6	0.07	39.0	**0.014**	35	42
Physicians	Drivers/Workforce	0.19	3.6	0.05	52.0	**0.001**	44	55

lmer(Linear trend ~ Action + Baseline + (1|income))
