# Supplementary material for: Association between national action and trends in antibiotic resistance: an analysis of 73 countries from 2000 to 2023
Source: PLOS Glob Public Health. 2025 Apr 30;5(4):e0004127. doi: 10.1371/journal.pgph.0004127 (PMC12043137; doi:10.1371/journal.pgph.0004127)
Supplement: S13 Table — (PDF) [file pgph.0004127.s020.pdf]

S13 Table. Association Between Categorical Trend and Action

Indicators	DPSE	Coefficient	t-value	std.error	df	p.value	Number of Countries with Increase	Sample Size
level 1								
Drivers Total	Drivers	-0.37	-1.4	0.27	69.1	0.169	6	73
Use Total	Use	-0.65	-3.2	0.21	61.0	**0.002**	55	65
Resistance Total	Resistance	-0.69	-2.9	0.24	29.0	**0.006**	16	32
DRI	DRI	-0.80	-2.1	0.38	22.0	**0.047**	21	25
level 2								
Infections	Drivers	0.18	0.9	0.20	69.2	0.364	12	73
Sanitation	Drivers	0.24	1.4	0.17	69.0	0.156	27	73
Vaccination	Drivers	-0.08	-0.4	0.21	69.1	0.693	11	73
Workforce	Drivers	-0.59	-2.8	0.21	51.2	**0.007**	9	55
TotalDDDPer1000Persons	Use	-0.01	-0.1	0.21	61.1	0.951	50	65
BroadPerTotalABXUse	Use	-0.55	-3.3	0.17	61.0	**0.001**	47	65
NewABXUse	Use	-0.60	-2.4	0.25	59.0	**0.02**	55	63
MRSA	Resistance	0.01	0.0	0.32	28.5	0.981	11	32
CR	Resistance	-0.38	-1.1	0.35	24.8	0.279	20	28
STR	Resistance	-0.62	-2.1	0.30	21.9	**0.049**	13	25
level 3								
HIV	Drivers/infections	-0.16	-0.7	0.23	27.0	0.486	22	31
TB	Drivers/infections	0.19	0.9	0.21	69.1	0.351	11	73
Drinking Water Source	Drivers/Sanitation	0.53	2.1	0.25	68.8	**0.038**	65	72
Water Source Access	Drivers/Sanitation	0.55	2.2	0.25	68.8	**0.033**	65	72
Overall Sanitation	Drivers/Sanitation	-0.29	-0.7	0.39	62.5	0.459	63	66
DTP3	Drivers/Vaccination	0.00	0.0	0.17	68.1	0.978	51	72
HepB3	Drivers/Vaccination	0.14	0.7	0.20	56.0	0.492	48	60
Hib3	Drivers/Vaccination	-0.26	-1.0	0.25	49.2	0.306	45	53
Pol3	Drivers/Vaccination	0.25	1.5	0.16	68.0	0.133	49	72
Measles	Drivers/Vaccination	0.11	0.7	0.17	69.1	0.503	53	73
RCV1	Drivers/Vaccination	0.15	0.8	0.19	58.3	0.428	43	62
Nursing	Drivers/Workforce	0.85	4.0	0.21	38.0	**< .001**	35	42
Physicians	Drivers/Workforce	0.54	2.7	0.20	51.0	**0.008**	44	55

lmer(Action ~ Categorical Trend + Baseline + (1|income))
