# Supplementary material for: Association between national action and trends in antibiotic resistance: an analysis of 73 countries from 2000 to 2023
Source: PLOS Glob Public Health. 2025 Apr 30;5(4):e0004127. doi: 10.1371/journal.pgph.0004127 (PMC12043137; doi:10.1371/journal.pgph.0004127)
Supplement: S14 Table — (PDF) [file pgph.0004127.s021.pdf]

S14 Table. Association Between Baseline and Action

Action is calculated from the mean of all action categories (See S7 Table). Association is determined with linear mixed model between baseline values for variables as a response variable and action as a fixed effect and income level as a random effect.

Indicators	DPSE	Coefficient	t-value	std.error	df	p.value	Number of Countries with Increase	Sample Size
level 1								
Drivers Total	Drivers	0.12	1.5	0.08	67.6	0.131	6	73
Use Total	Use	0.00	0.0	0.13	63.0	0.982	55	65
Resistance Total	Resistance	-0.16	-1.1	0.14	30.0	0.265	16	32
DRI	DRI	-0.30	-1.6	0.19	22.9	0.126	21	25
level 2								
Infections	Drivers	0.02	0.2	0.09	69.8	0.835	12	73
Sanitation	Drivers	0.09	1.2	0.08	71.0	0.244	27	73
Vaccination	Drivers	0.18	1.3	0.14	41.1	0.216	11	73
Workforce	Drivers	0.19	1.4	0.14	52.8	0.182	9	55
TotalDDDPer1000Persons	Use	-0.12	-0.8	0.15	63.0	0.442	50	65
BroadPerTotalABXUse	Use	0.03	0.2	0.17	62.4	0.859	47	65
NewABXUse	Use	0.14	0.8	0.17	57.0	0.405	55	63
MRSA	Resistance	-0.21	-0.8	0.26	26.4	0.423	11	32
CR	Resistance	-0.06	-0.6	0.11	26.0	0.569	20	28
STR	Resistance	-0.13	-0.6	0.20	23.0	0.529	13	25
level 3								
HIV	Drivers/infections	0.03	0.1	0.19	29.0	0.887	22	31
TB	Drivers/infections	-0.01	-0.1	0.11	70.8	0.919	11	73
Drinking Water Source	Drivers/Sanitation	0.04	0.7	0.06	69.8	0.46	65	72
Water Source Access	Drivers/Sanitation	0.07	1.2	0.06	69.9	0.25	65	72
Overall Sanitation	Drivers/Sanitation	0.10	1.0	0.10	64.0	0.323	63	66
DTP3	Drivers/Vaccination	0.06	0.6	0.09	56.8	0.529	51	72
HepB3	Drivers/Vaccination	0.06	0.3	0.23	58.0	0.802	48	60
Hib3	Drivers/Vaccination	0.43	2.6	0.17	11.3	**0.026**	45	53
Pol3	Drivers/Vaccination	0.07	0.7	0.10	51.4	0.494	49	72
Measles	Drivers/Vaccination	0.09	1.0	0.09	71.0	0.335	53	73
RCV1	Drivers/Vaccination	-0.03	-0.2	0.14	60.0	0.82	43	62
Nursing	Drivers/Workforce	0.27	1.4	0.19	39.7	0.163	35	42
Physicians	Drivers/Workforce	0.18	1.1	0.16	52.9	0.273	44	55

lmer(Baseline ~ Action + (1|income))
