# Supplementary material for: Association between national action and trends in antibiotic resistance: an analysis of 73 countries from 2000 to 2023
Source: PLOS Glob Public Health. 2025 Apr 30;5(4):e0004127. doi: 10.1371/journal.pgph.0004127 (PMC12043137; doi:10.1371/journal.pgph.0004127)
Supplement: S15 Table — (PDF) [file pgph.0004127.s022.pdf]

S15 Table. Linear Trend and Awareness and Education

Indicators	DPSE	Coefficient	t-value	std.error	df	p.value	Number of Countries with Increase	Sample Size
level 1								
Drivers Total	Drivers	-0.04	-2.0	0.02	70.0	**0.046**	6	73
Use Total	Use	-0.08	-1.3	0.06	62.0	0.205	55	65
Resistance Total	Resistance	-0.04	-0.4	0.09	29.0	0.666	16	32
DRI	DRI	-0.12	-1.7	0.07	21.2	0.113	21	25
level 2								
Infections	Drivers	0.01	0.5	0.01	69.9	0.593	12	73
Sanitation	Drivers	-0.02	-1.6	0.01	69.8	0.12	27	73
Vaccination	Drivers	-0.06	-1.3	0.05	70.0	0.201	11	73
Workforce	Drivers	-0.10	-2.1	0.05	52.0	**0.04**	9	55
TotalDDDPer1000Persons	Use	0.08	1.0	0.08	61.6	0.305	50	65
BroadPerTotalABXUse	Use	-0.17	-2.1	0.08	62.0	**0.039**	47	65
NewABXUse	Use	-0.15	-2.1	0.07	60.0	**0.043**	55	63
MRSA	Resistance	-0.08	-0.8	0.10	29.0	0.433	11	32
CR	Resistance	0.04	0.2	0.19	24.8	0.831	20	28
STR	Resistance	-0.11	-1.7	0.07	21.7	0.106	13	25
level 3								
HIV	Drivers/infections	0.02	2.2	0.01	28.0	**0.034**	22	31
TB	Drivers/infections	0.00	0.2	0.02	69.3	0.82	11	73
Drinking Water Source	Drivers/Sanitation	0.02	1.4	0.02	69.0	0.181	65	72
Water Source Access	Drivers/Sanitation	0.02	1.4	0.01	69.0	0.168	65	72
Overall Sanitation	Drivers/Sanitation	0.02	1.2	0.01	62.6	0.234	63	66
DTP3	Drivers/Vaccination	0.07	1.2	0.06	54.9	0.251	51	72
HepB3	Drivers/Vaccination	0.11	1.5	0.07	57.0	0.151	48	60
Hib3	Drivers/Vaccination	0.07	1.2	0.06	47.6	0.245	45	53
Pol3	Drivers/Vaccination	0.08	1.6	0.05	47.3	0.124	49	72
Measles	Drivers/Vaccination	0.08	1.8	0.05	70.0	0.082	53	73
RCV1	Drivers/Vaccination	0.13	1.7	0.07	59.0	0.094	43	62
Nursing	Drivers/Workforce	0.06	1.0	0.06	39.0	0.307	35	42
Physicians	Drivers/Workforce	0.11	2.4	0.05	52.0	**0.019**	44	55

lmer(Linear Trend ~ Awareness and Education + Baseline + (1|income))
