# Supplementary material for: Association between national action and trends in antibiotic resistance: an analysis of 73 countries from 2000 to 2023
Source: PLOS Glob Public Health. 2025 Apr 30;5(4):e0004127. doi: 10.1371/journal.pgph.0004127 (PMC12043137; doi:10.1371/journal.pgph.0004127)
Supplement: S16 Table — (PDF) [file pgph.0004127.s023.pdf]

S16 Table. Categorical Trend and Awareness and Education

Indicators	DPSE	Coefficient	t-value	std.error	df	p.value	Number of Countries with Increase	Sample Size
level 1								
Drivers Total	Drivers	-0.43	-1.3	0.34	69.3	0.211	6	73
Use Total	Use	-0.66	-2.6	0.26	61.0	**0.013**	55	65
Resistance Total	Resistance	-0.66	-2.4	0.27	29.0	**0.022**	16	32
DRI	DRI	-0.79	-1.8	0.43	21.9	0.08	21	25
level 2								
Infections	Drivers	0.22	0.9	0.25	69.6	0.388	12	73
Sanitation	Drivers	0.24	1.1	0.21	69.2	0.27	27	73
Vaccination	Drivers	-0.27	-1.0	0.26	69.2	0.312	11	73
Workforce	Drivers	-0.62	-2.2	0.29	51.8	**0.033**	9	55
TotalDDDPer1000Persons	Use	0.15	0.6	0.25	61.5	0.563	50	65
BroadPerTotalABXUse	Use	-0.53	-2.5	0.21	61.0	**0.016**	47	65
NewABXUse	Use	-0.63	-2.1	0.31	59.0	**0.045**	55	63
MRSA	Resistance	-0.03	-0.1	0.36	28.7	0.924	11	32
CR	Resistance	-0.53	-1.4	0.37	25.0	0.168	20	28
STR	Resistance	-0.82	-2.3	0.35	21.5	**0.029**	13	25
level 3								
HIV	Drivers/infections	0.14	0.4	0.34	27.0	0.681	22	31
TB	Drivers/infections	0.19	0.8	0.26	69.3	0.453	11	73
Drinking Water Source	Drivers/Sanitation	0.78	2.6	0.30	69.0	**0.011**	65	72
Water Source Access	Drivers/Sanitation	0.69	2.2	0.31	63.2	**0.03**	65	72
Overall Sanitation	Drivers/Sanitation	-0.14	-0.3	0.49	62.8	0.768	63	66
DTP3	Drivers/Vaccination	0.08	0.4	0.21	68.4	0.687	51	72
HepB3	Drivers/Vaccination	0.18	0.7	0.25	56.0	0.468	48	60
Hib3	Drivers/Vaccination	-0.04	-0.1	0.33	49.4	0.891	45	53
Pol3	Drivers/Vaccination	0.15	0.7	0.20	68.1	0.458	49	72
Measles	Drivers/Vaccination	0.03	0.1	0.21	69.4	0.886	53	73
RCV1	Drivers/Vaccination	0.02	0.1	0.24	58.8	0.925	43	62
Nursing	Drivers/Workforce	0.89	2.9	0.30	38.0	**0.006**	35	42
Physicians	Drivers/Workforce	0.41	1.5	0.27	51.0	0.135	44	55

lmer(Awareness and Education ~ Categorical Trend + Baseline + (1|income))
