# Supplementary material for: Association between national action and trends in antibiotic resistance: an analysis of 73 countries from 2000 to 2023
Source: PLOS Glob Public Health. 2025 Apr 30;5(4):e0004127. doi: 10.1371/journal.pgph.0004127 (PMC12043137; doi:10.1371/journal.pgph.0004127)
Supplement: S17 Table — (PDF) [file pgph.0004127.s024.pdf]

S17 Table. Linear Trend and General

Indicators	DPSE	Coefficient	t-value	std.error	df	p.value	Number of Countries with Increase	Sample Size
level 1								
Drivers Total	Drivers	-0.03	-1.8	0.02	70.0	0.078	6	73
Use Total	Use	-0.09	-1.9	0.05	61.7	0.066	55	65
Resistance Total	Resistance	-0.11	-1.7	0.06	29.0	0.097	16	32
DRI	DRI	-0.12	-2.4	0.05	21.1	**0.026**	21	25
level 2								
Infections	Drivers	0.02	2.0	0.01	62.6	**0.045**	12	73
Sanitation	Drivers	-0.01	-0.4	0.01	69.2	0.674	27	73
Vaccination	Drivers	-0.03	-0.8	0.04	38.2	0.432	11	73
Workforce	Drivers	-0.14	-3.4	0.04	52.0	**0.001**	9	55
TotalDDDPer1000Persons	Use	0.04	0.7	0.07	61.9	0.512	50	65
BroadPerTotalABXUse	Use	-0.19	-2.9	0.07	62.0	**0.005**	47	65
NewABXUse	Use	-0.11	-1.7	0.06	60.0	0.091	55	63
MRSA	Resistance	-0.14	-2.0	0.07	29.0	0.06	11	32
CR	Resistance	-0.13	-1.0	0.13	25.0	0.309	20	28
STR	Resistance	-0.06	-1.2	0.05	22.0	0.229	13	25
level 3								
HIV	Drivers/infections	0.02	2.2	0.01	28.0	**0.036**	22	31
TB	Drivers/infections	0.03	1.9	0.02	43.4	0.061	11	73
Drinking Water Source	Drivers/Sanitation	0.01	0.6	0.01	69.0	0.582	65	72
Water Source Access	Drivers/Sanitation	0.00	0.4	0.01	69.0	0.713	65	72
Overall Sanitation	Drivers/Sanitation	0.01	0.8	0.01	63.0	0.413	63	66
DTP3	Drivers/Vaccination	0.05	1.0	0.05	43.1	0.334	51	72
HepB3	Drivers/Vaccination	0.10	1.4	0.07	57.0	0.168	48	60
Hib3	Drivers/Vaccination	-0.07	-1.2	0.06	49.8	0.228	45	53
Pol3	Drivers/Vaccination	0.07	1.4	0.05	32.5	0.163	49	72
Measles	Drivers/Vaccination	0.07	1.7	0.04	70.0	0.094	53	73
RCV1	Drivers/Vaccination	0.11	1.9	0.06	59.0	0.068	43	62
Nursing	Drivers/Workforce	0.15	2.7	0.06	39.0	**0.011**	35	42
Physicians	Drivers/Workforce	0.13	3.1	0.04	52.0	**0.003**	44	55

lmer(Linear Trend ~ General + Baseline + (1|income))
