# Supplementary material for: Association between national action and trends in antibiotic resistance: an analysis of 73 countries from 2000 to 2023
Source: PLOS Glob Public Health. 2025 Apr 30;5(4):e0004127. doi: 10.1371/journal.pgph.0004127 (PMC12043137; doi:10.1371/journal.pgph.0004127)
Supplement: S18 Table — (PDF) [file pgph.0004127.s025.pdf]

S18 Table. Categorical Trend and General

Indicators	DPSE	Coefficient	t-value	std.error	df	p.value	Number of Countries with Increase	Sample Size
level 1								
Drivers Total	Drivers	-0.59	-1.5	0.39	69.2	0.133	6	73
Use Total	Use	-0.84	-2.8	0.30	61.0	**0.008**	55	65
Resistance Total	Resistance	-1.13	-3.2	0.35	29.0	**0.003**	16	32
DRI	DRI	-1.08	-1.8	0.58	22.0	0.078	21	25
level 2								
Infections	Drivers	0.55	2.0	0.28	69.4	0.05	12	73
Sanitation	Drivers	0.40	1.6	0.24	69.1	0.107	27	73
Vaccination	Drivers	-0.01	0.0	0.30	69.2	0.982	11	73
Workforce	Drivers	-0.99	-3.4	0.29	51.4	**0.001**	9	55
TotalDDDPer1000Persons	Use	0.23	0.8	0.30	61.3	0.435	50	65
BroadPerTotalABXUse	Use	-0.67	-2.7	0.25	61.0	**0.009**	47	65
NewABXUse	Use	-0.79	-2.1	0.37	59.0	**0.036**	55	63
MRSA	Resistance	0.29	0.6	0.48	28.5	0.551	11	32
CR	Resistance	-0.52	-0.9	0.55	24.8	0.356	20	28
STR	Resistance	-0.76	-1.6	0.48	21.9	0.129	13	25
level 3								
HIV	Drivers/infections	0.19	0.6	0.30	27.0	0.533	22	31
TB	Drivers/infections	0.67	2.4	0.28	69.2	**0.02**	11	73
Drinking Water Source	Drivers/Sanitation	1.00	2.8	0.35	68.8	**0.006**	65	72
Water Source Access	Drivers/Sanitation	0.96	2.7	0.36	68.9	**0.009**	65	72
Overall Sanitation	Drivers/Sanitation	-0.32	-0.6	0.56	62.8	0.573	63	66
DTP3	Drivers/Vaccination	0.05	0.2	0.24	68.3	0.841	51	72
HepB3	Drivers/Vaccination	0.20	0.8	0.26	56.0	0.436	48	60
Hib3	Drivers/Vaccination	-0.73	-2.1	0.36	49.6	**0.045**	45	53
Pol3	Drivers/Vaccination	0.41	1.8	0.23	68.0	0.076	49	72
Measles	Drivers/Vaccination	0.26	1.1	0.24	69.4	0.294	53	73
RCV1	Drivers/Vaccination	0.42	1.5	0.28	58.7	0.135	43	62
Nursing	Drivers/Workforce	1.24	4.3	0.29	39.0	**< .001**	35	42
Physicians	Drivers/Workforce	0.61	2.2	0.28	51.0	**0.036**	44	55

lmer(General ~ Categorical Trend + Baseline + (1|income))
