# Supplementary material for: Association between national action and trends in antibiotic resistance: an analysis of 73 countries from 2000 to 2023
Source: PLOS Glob Public Health. 2025 Apr 30;5(4):e0004127. doi: 10.1371/journal.pgph.0004127 (PMC12043137; doi:10.1371/journal.pgph.0004127)
Supplement: S19 Table — (PDF) [file pgph.0004127.s026.pdf]

S19 Table. Linear Trend and Monitoring and Surveillance

Indicators	DPSE	Coefficient	t-value	std.error	df	p.value	Number of Countries with Increase	Sample Size
level 1								
Drivers Total	Drivers	-0.06	-3.1	0.02	39.1	**0.003**	6	73
Use Total	Use	-0.19	-4.1	0.05	62.0	**< .001**	55	65
Resistance Total	Resistance	-0.15	-1.1	0.13	18.7	0.281	16	32
DRI	DRI	-0.14	-1.1	0.13	21.1	0.278	21	25
level 2								
Infections	Drivers	0.00	0.1	0.01	39.2	0.896	12	73
Sanitation	Drivers	0.00	-0.2	0.02	52.7	0.862	27	73
Vaccination	Drivers	-0.07	-1.6	0.04	70.0	0.112	11	73
Workforce	Drivers	-0.15	-2.9	0.05	52.0	**0.006**	9	55
TotalDDDPer1000Persons	Use	-0.09	-1.1	0.08	49.8	0.286	50	65
BroadPerTotalABXUse	Use	-0.25	-3.1	0.08	39.1	**0.004**	47	65
NewABXUse	Use	-0.14	-2.3	0.06	60.0	**0.023**	55	63
MRSA	Resistance	-0.13	-0.9	0.15	29.0	0.386	11	32
CR	Resistance	-0.30	-1.3	0.23	25.0	0.214	20	28
STR	Resistance	-0.30	-3.5	0.09	22.0	**0.002**	13	25
level 3								
HIV	Drivers/infections	0.01	0.8	0.01	28.0	0.419	22	31
TB	Drivers/infections	0.02	1.3	0.02	6.4	0.228	11	73
Drinking Water Source	Drivers/Sanitation	0.01	0.4	0.02	69.0	0.71	65	72
Water Source Access	Drivers/Sanitation	0.01	0.5	0.01	69.0	0.601	65	72
Overall Sanitation	Drivers/Sanitation	0.00	0.1	0.01	55.5	0.955	63	66
DTP3	Drivers/Vaccination	0.07	1.6	0.05	69.0	0.124	51	72
HepB3	Drivers/Vaccination	0.06	0.9	0.07	57.0	0.379	48	60
Hib3	Drivers/Vaccination	0.11	2.1	0.05	50.0	**0.042**	45	53
Pol3	Drivers/Vaccination	0.08	1.8	0.04	69.0	0.071	49	72
Measles	Drivers/Vaccination	0.07	1.8	0.04	70.0	0.08	53	73
RCV1	Drivers/Vaccination	0.12	1.9	0.06	59.0	0.064	43	62
Nursing	Drivers/Workforce	0.13	1.8	0.07	39.0	0.074	35	42
Physicians	Drivers/Workforce	0.12	2.6	0.05	52.0	**0.011**	44	55

lmer(Linear Trend ~ Monitoring and Surveillance + Baseline + (1|income))
