# Supplementary material for: Association between national action and trends in antibiotic resistance: an analysis of 73 countries from 2000 to 2023
Source: PLOS Glob Public Health. 2025 Apr 30;5(4):e0004127. doi: 10.1371/journal.pgph.0004127 (PMC12043137; doi:10.1371/journal.pgph.0004127)
Supplement: S20 Table — (PDF) [file pgph.0004127.s027.pdf]

S20 Table. Categorical Trend and Monitoring and Surveillance

Indicators	DPSE	Coefficient	t-value	std.error	df	p.value	Number of Countries with Increase	Sample Size
level 1								
Drivers Total	Drivers	-0.09	-0.3	0.30	69.0	0.752	6	73
Use Total	Use	-0.61	-2.4	0.25	61.0	**0.019**	55	65
Resistance Total	Resistance	-0.47	-2.6	0.18	28.2	**0.013**	16	32
DRI	DRI	-0.12	-0.4	0.27	20.5	0.671	21	25
level 2								
Infections	Drivers	-0.05	-0.2	0.24	69.1	0.83	12	73
Sanitation	Drivers	0.45	2.3	0.19	69.0	**0.022**	27	73
Vaccination	Drivers	-0.06	-0.2	0.24	69.0	0.822	11	73
Workforce	Drivers	-0.57	-2.4	0.24	51.2	**0.019**	9	55
TotalDDDPer1000Persons	Use	-0.28	-1.2	0.25	61.1	0.253	50	65
BroadPerTotalABXUse	Use	-0.58	-2.8	0.20	61.0	**0.006**	47	65
NewABXUse	Use	-0.43	-1.4	0.31	59.0	0.164	55	63
MRSA	Resistance	0.03	0.1	0.23	28.4	0.902	11	32
CR	Resistance	-0.53	-2.0	0.27	24.6	0.058	20	28
STR	Resistance	-0.54	-2.4	0.23	22.0	**0.025**	13	25
level 3								
HIV	Drivers/infections	-0.30	-1.1	0.27	27.0	0.282	22	31
TB	Drivers/infections	-0.02	-0.1	0.25	69.0	0.939	11	73
Drinking Water Source	Drivers/Sanitation	0.32	1.1	0.30	68.5	0.285	65	72
Water Source Access	Drivers/Sanitation	0.35	1.2	0.30	68.5	0.243	65	72
Overall Sanitation	Drivers/Sanitation	0.02	0.1	0.44	62.3	0.957	63	66
DTP3	Drivers/Vaccination	-0.19	-1.0	0.19	68.1	0.34	51	72
HepB3	Drivers/Vaccination	-0.14	-0.6	0.24	56.0	0.557	48	60
Hib3	Drivers/Vaccination	-0.26	-0.8	0.31	49.1	0.401	45	53
Pol3	Drivers/Vaccination	-0.05	-0.3	0.19	68.0	0.785	49	72
Measles	Drivers/Vaccination	0.00	0.0	0.20	69.1	1	53	73
RCV1	Drivers/Vaccination	0.13	0.6	0.22	58.2	0.564	43	62
Nursing	Drivers/Workforce	0.75	2.9	0.26	38.0	**0.006**	35	42
Physicians	Drivers/Workforce	0.48	2.0	0.24	51.0	**0.048**	44	55

lmer(Monitoring and Surveillance ~ Categorical Trend + Baseline + (1|income))
