# Supplementary material for: Association between national action and trends in antibiotic resistance: an analysis of 73 countries from 2000 to 2023
Source: PLOS Glob Public Health. 2025 Apr 30;5(4):e0004127. doi: 10.1371/journal.pgph.0004127 (PMC12043137; doi:10.1371/journal.pgph.0004127)
Supplement: S21 Table — (PDF) [file pgph.0004127.s028.pdf]

S21 Table. Linear Trend and Prevention

Indicators	DPSE	Coefficient	t-value	std.error	df	p.value	Number of Countries with Increase	Sample Size
level 1								
Drivers Total	Drivers	-0.01	-0.4	0.02	70.0	0.661	6	73
Use Total	Use	-0.02	-0.5	0.05	62.0	0.642	55	65
Resistance Total	Resistance	-0.06	-1.0	0.06	27.7	0.339	16	32
DRI	DRI	-0.09	-1.9	0.05	21.0	0.078	21	25
level 2								
Infections	Drivers	0.01	1.0	0.01	70.0	0.324	12	73
Sanitation	Drivers	0.02	1.7	0.01	70.0	0.096	27	73
Vaccination	Drivers	-0.04	-0.9	0.04	63.3	0.383	11	73
Workforce	Drivers	-0.08	-2.2	0.04	52.0	**0.035**	9	55
TotalDDDPer1000Persons	Use	0.03	0.5	0.07	62.0	0.65	50	65
BroadPerTotalABXUse	Use	-0.09	-1.3	0.07	62.0	0.211	47	65
NewABXUse	Use	0.01	0.2	0.06	58.4	0.822	55	63
MRSA	Resistance	-0.10	-1.4	0.07	29.0	0.173	11	32
CR	Resistance	-0.07	-0.6	0.13	21.4	0.583	20	28
STR	Resistance	-0.10	-1.7	0.06	22.0	0.106	13	25
level 3								
HIV	Drivers/infections	0.01	0.7	0.01	28.0	0.486	22	31
TB	Drivers/infections	0.01	0.5	0.02	70.0	0.64	11	73
Drinking Water Source	Drivers/Sanitation	-0.02	-1.4	0.01	69.0	0.153	65	72
Water Source Access	Drivers/Sanitation	-0.02	-1.4	0.01	69.0	0.158	65	72
Overall Sanitation	Drivers/Sanitation	0.01	0.7	0.01	62.3	0.506	63	66
DTP3	Drivers/Vaccination	0.05	1.0	0.05	61.6	0.324	51	72
HepB3	Drivers/Vaccination	0.08	1.3	0.06	57.0	0.212	48	60
Hib3	Drivers/Vaccination	-0.01	-0.2	0.06	49.4	0.829	45	53
Pol3	Drivers/Vaccination	0.08	1.8	0.04	50.4	0.084	49	72
Measles	Drivers/Vaccination	0.04	1.0	0.04	58.7	0.346	53	73
RCV1	Drivers/Vaccination	0.06	0.9	0.06	56.7	0.385	43	62
Nursing	Drivers/Workforce	0.07	1.3	0.05	39.0	0.193	35	42
Physicians	Drivers/Workforce	0.08	2.0	0.04	52.0	**0.046**	44	55

lmer(Linear Trend ~ Prevention + Baseline + (1|income))
