# Supplementary material for: Association between national action and trends in antibiotic resistance: an analysis of 73 countries from 2000 to 2023
Source: PLOS Glob Public Health. 2025 Apr 30;5(4):e0004127. doi: 10.1371/journal.pgph.0004127 (PMC12043137; doi:10.1371/journal.pgph.0004127)
Supplement: S22 Table — (PDF) [file pgph.0004127.s029.pdf]

S22 Table. Categorical Trend and Prevention

Indicators	DPSE	Coefficient	t-value	std.error	df	p.value	Number of Countries with Increase	Sample Size
level 1								
Drivers Total	Drivers	-0.11	-0.3	0.42	69.3	0.796	6	73
Use Total	Use	-0.54	-1.6	0.33	61.0	0.107	55	65
Resistance Total	Resistance	-0.67	-1.7	0.39	24.1	0.096	16	32
DRI	DRI	-0.80	-1.3	0.62	22.0	0.209	21	25
level 2								
Infections	Drivers	-0.03	-0.1	0.30	69.7	0.926	12	73
Sanitation	Drivers	0.12	0.5	0.26	69.2	0.647	27	73
Vaccination	Drivers	0.08	0.3	0.31	69.3	0.802	11	73
Workforce	Drivers	-0.35	-1.0	0.36	51.3	0.335	9	55
TotalDDDPer1000Persons	Use	-0.11	-0.4	0.31	61.3	0.718	50	65
BroadPerTotalABXUse	Use	-0.37	-1.4	0.27	61.0	0.18	47	65
NewABXUse	Use	-0.33	-0.8	0.39	59.0	0.4	55	63
MRSA	Resistance	0.19	0.4	0.48	28.6	0.69	11	32
CR	Resistance	-0.27	-0.5	0.55	24.8	0.627	20	28
STR	Resistance	-0.63	-1.5	0.43	21.8	0.158	13	25
level 3								
HIV	Drivers/infections	-0.42	-1.3	0.32	27.0	0.202	22	31
TB	Drivers/infections	-0.07	-0.2	0.31	69.4	0.835	11	73
Drinking Water Source	Drivers/Sanitation	0.18	0.5	0.39	68.3	0.644	65	72
Water Source Access	Drivers/Sanitation	0.46	1.2	0.37	69.0	0.223	65	72
Overall Sanitation	Drivers/Sanitation	-0.20	-0.4	0.51	59.0	0.703	63	66
DTP3	Drivers/Vaccination	0.02	0.1	0.25	68.4	0.952	51	72
HepB3	Drivers/Vaccination	0.38	1.2	0.31	56.0	0.233	48	60
Hib3	Drivers/Vaccination	-0.22	-0.6	0.36	49.7	0.544	45	53
Pol3	Drivers/Vaccination	0.37	1.5	0.24	68.1	0.135	49	72
Measles	Drivers/Vaccination	0.02	0.1	0.26	69.5	0.931	53	73
RCV1	Drivers/Vaccination	-0.01	0.0	0.30	59.0	0.965	43	62
Nursing	Drivers/Workforce	0.61	1.7	0.36	38.0	0.101	35	42
Physicians	Drivers/Workforce	0.74	2.3	0.32	51.0	**0.023**	44	55

lmer(Prevention ~ Categorical Trend + Baseline + (1|income))
