# Supplementary material for: Association between national action and trends in antibiotic resistance: an analysis of 73 countries from 2000 to 2023
Source: PLOS Glob Public Health. 2025 Apr 30;5(4):e0004127. doi: 10.1371/journal.pgph.0004127 (PMC12043137; doi:10.1371/journal.pgph.0004127)
Supplement: S23 Table — (PDF) [file pgph.0004127.s030.pdf]

S23 Table. Linear Trend and Regulation

Indicators	DPSE	Coefficient	t-value	std.error	df	p.value	Number of Countries with Increase	Sample Size
level 1								
Drivers Total	Drivers	-0.04	-2.8	0.01	26.0	**0.01**	6	73
Use Total	Use	-0.08	-1.5	0.05	60.6	0.129	55	65
Resistance Total	Resistance	-0.01	-0.1	0.10	28.2	0.946	16	32
DRI	DRI	-0.10	-1.3	0.08	21.1	0.207	21	25
level 2								
Infections	Drivers	-0.01	-0.8	0.01	68.5	0.405	12	73
Sanitation	Drivers	-0.02	-1.9	0.01	69.8	0.058	27	73
Vaccination	Drivers	-0.06	-1.6	0.04	70.0	0.121	11	73
Workforce	Drivers	-0.12	-3.1	0.04	52.0	**0.003**	9	55
TotalDDDPer1000Persons	Use	0.02	0.3	0.07	62.0	0.758	50	65
BroadPerTotalABXUse	Use	-0.17	-2.4	0.07	20.4	**0.026**	47	65
NewABXUse	Use	-0.07	-1.1	0.06	60.0	0.258	55	63
MRSA	Resistance	-0.13	-1.2	0.11	29.0	0.255	11	32
CR	Resistance	0.09	0.4	0.21	24.5	0.677	20	28
STR	Resistance	0.04	0.5	0.08	21.4	0.636	13	25
level 3								
HIV	Drivers/infections	0.00	0.6	0.01	28.0	0.555	22	31
TB	Drivers/infections	0.00	0.0	0.02	61.8	0.97	11	73
Drinking Water Source	Drivers/Sanitation	0.02	1.6	0.01	49.5	0.106	65	72
Water Source Access	Drivers/Sanitation	0.02	1.8	0.01	44.4	0.086	65	72
Overall Sanitation	Drivers/Sanitation	0.00	0.1	0.01	61.5	0.933	63	66
DTP3	Drivers/Vaccination	0.08	1.8	0.04	69.0	0.08	51	72
HepB3	Drivers/Vaccination	0.04	0.6	0.06	57.0	0.529	48	60
Hib3	Drivers/Vaccination	0.01	0.1	0.06	44.0	0.902	45	53
Pol3	Drivers/Vaccination	0.08	2.0	0.04	69.0	**0.046**	49	72
Measles	Drivers/Vaccination	0.10	2.7	0.03	70.0	**0.008**	53	73
RCV1	Drivers/Vaccination	0.11	1.9	0.06	59.0	0.057	43	62
Nursing	Drivers/Workforce	0.13	2.5	0.05	39.0	**0.015**	35	42
Physicians	Drivers/Workforce	0.11	2.7	0.04	52.0	**0.009**	44	55

lmer(Linear Trend ~ Regulation + Baseline + (1|income))
