# Supplementary material for: Association between national action and trends in antibiotic resistance: an analysis of 73 countries from 2000 to 2023
Source: PLOS Glob Public Health. 2025 Apr 30;5(4):e0004127. doi: 10.1371/journal.pgph.0004127 (PMC12043137; doi:10.1371/journal.pgph.0004127)
Supplement: S24 Table — (PDF) [file pgph.0004127.s031.pdf]

S24 Table. Categorical Trend and Regulation

Indicators	DPSE	Coefficient	t-value	std.error	df	p.value	Number of Countries with Increase	Sample Size
level 1								
Drivers Total	Drivers	-0.61	-1.6	0.39	69.1	0.122	6	73
Use Total	Use	-0.60	-2.0	0.30	61.0	0.051	55	65
Resistance Total	Resistance	-0.43	-1.6	0.26	29.0	0.115	16	32
DRI	DRI	-1.04	-2.8	0.38	21.9	**0.012**	21	25
level 2								
Infections	Drivers	0.27	0.9	0.29	69.2	0.363	12	73
Sanitation	Drivers	0.03	0.1	0.25	69.1	0.91	27	73
Vaccination	Drivers	-0.19	-0.6	0.30	69.1	0.532	11	73
Workforce	Drivers	-0.37	-1.1	0.34	51.4	0.285	9	55
TotalDDDPer1000Persons	Use	-0.08	-0.3	0.29	61.3	0.773	50	65
BroadPerTotalABXUse	Use	-0.62	-2.6	0.24	61.0	**0.012**	47	65
NewABXUse	Use	-0.82	-2.3	0.35	59.0	**0.023**	55	63
MRSA	Resistance	-0.37	-1.2	0.32	29.0	0.256	11	32
CR	Resistance	-0.17	-0.5	0.35	25.0	0.643	20	28
STR	Resistance	-0.50	-1.5	0.32	22.0	0.139	13	25
level 3								
HIV	Drivers/infections	-0.41	-1.2	0.35	27.0	0.246	22	31
TB	Drivers/infections	0.22	0.7	0.30	69.1	0.473	11	73
Drinking Water Source	Drivers/Sanitation	0.51	1.4	0.37	68.8	0.179	65	72
Water Source Access	Drivers/Sanitation	0.43	1.1	0.37	68.8	0.255	65	72
Overall Sanitation	Drivers/Sanitation	-0.64	-1.2	0.55	62.5	0.253	63	66
DTP3	Drivers/Vaccination	0.09	0.4	0.24	68.1	0.712	51	72
HepB3	Drivers/Vaccination	0.06	0.2	0.31	56.0	0.847	48	60
Hib3	Drivers/Vaccination	0.03	0.1	0.34	49.2	0.94	45	53
Pol3	Drivers/Vaccination	0.36	1.5	0.24	68.0	0.132	49	72
Measles	Drivers/Vaccination	0.29	1.2	0.25	69.2	0.236	53	73
RCV1	Drivers/Vaccination	0.27	1.0	0.27	58.4	0.336	43	62
Nursing	Drivers/Workforce	0.76	2.1	0.36	38.0	**0.041**	35	42
Physicians	Drivers/Workforce	0.47	1.5	0.31	51.0	0.135	44	55

lmer(Regulation ~ Categorical Trend + Baseline + (1|income))
