# Supplementary material for: Association between national action and trends in antibiotic resistance: an analysis of 73 countries from 2000 to 2023
Source: PLOS Glob Public Health. 2025 Apr 30;5(4):e0004127. doi: 10.1371/journal.pgph.0004127 (PMC12043137; doi:10.1371/journal.pgph.0004127)
Supplement: S25 Table — (PDF) [file pgph.0004127.s032.pdf]

S25 Table. De-escalation Merged Model Comparison Results.
Merged model comparison for de-escalation to investigate the importance of action for declining proportion, including all DPSE categories. df: residual degrees of freedom. AICc: Akaike information criterion corrected for small sample sizes. Delta AICc: Difference between the AICc of the best model and the given model. Weight is the AICc weight.

**Model Formula**	**df**	**logLik**	**AICc**	**Delta AICc**	**weight**
Declining proportion ~ Action * DPSE + Baseline Mean	186	-179.69	378.36	0	1
Declining proportion ~ Action + DPSE + Baseline Mean	189	-192.48	397.40	19.04	0
Declining proportion ~ Baseline Mean	193	-255.20	514.47	136.11	0
Declining proportion ~ Action + Baseline Mean	192	-255.14	516.40	138.04	0
