# Supplementary material for: Association between national action and trends in antibiotic resistance: an analysis of 73 countries from 2000 to 2023
Source: PLOS Glob Public Health. 2025 Apr 30;5(4):e0004127. doi: 10.1371/journal.pgph.0004127 (PMC12043137; doi:10.1371/journal.pgph.0004127)
Supplement: S26 Table — (PDF) [file pgph.0004127.s033.pdf]

S26 Table. De-escalation Merged Model Results.
Merged model results for de-escalation to investigate the importance of action. Model results derived from the merged model with the main formula as in S9 Table and reported for all the categories.

**Indicators**	**Odds Ratio**	**CI**	**P value**
Action (DRIVERS)	0.62	0.41 – 0.92	**0.018**
Action (USE-DRIVERS)	4.23	2.26 – 8.11	**<0.001**
Action (RESISTANCE-DRIVERS)	2.25	1.11 – 4.65	**0.026**
Action (DRI-DRIVERS)	13.53	1.87 – 317.75	**0.035**
