# Supplementary material for: Association between national action and trends in antibiotic resistance: an analysis of 73 countries from 2000 to 2023
Source: PLOS Glob Public Health. 2025 Apr 30;5(4):e0004127. doi: 10.1371/journal.pgph.0004127 (PMC12043137; doi:10.1371/journal.pgph.0004127)
Supplement: S27 Table — (PDF) [file pgph.0004127.s034.pdf]

S27 Table. Model selection table including variables for best selected models and null models.

Model selection summary tables included first 5 best selected models and the null model to determine the most important variables affecting Antibiotic Resistance (ABR) linear trend in 16 years. Two model types are included in the table. First model type used linear trend as a response variable and determining the change of ABR indicators between two intervals (2008-2016 vs 2000-2008). Second model type used categorical trend as a response variable, and it refers to the positive or negative change. Note that linear trend models run with different datasets including data from i. all countries, ii. High Income Countries, iii. Low- and Middle-Income Countries according to the income column. Overall, all models run with a specific data subset and named accordingly (see Fig 4 capture for model names). DPSEA refers to the indicators from the framework (see methods), Baseline refers to the Mean of DPSE Indicator in Baseline (2000−2008), income refers to the high income of low and middle income. For country names matching with ISO3 codes see S5 Table, for Tier 2 indicators and other variable abbreviations see S1-S4 Table. df =degrees of freedom. AICc: Akaike information criterion corrected for small sample sizes. Delta AICc: Difference between the AICc of the best model and the given model. Action variables are indicated in *italics*.

Model Name	Variables	df	logLik	AICc	delta	weight	income
**DPSEA**	Linear Trend ~ *General* + Baseline + DPSE * Workforce	12	-39.913	106.023	0.000	0.314	ALL
**DPSEA**	Linear Trend ~ *Action* + Baseline + DPSE * Workforce	12	-40.212	106.621	0.599	0.233	ALL
**DPSEA**	Linear Trend ~ Baseline + DPSE * Workforce	11	-41.480	106.807	0.784	0.212	ALL
**DPSEA**	Linear Trend ~ *Monitoring and Surveillance* + Baseline + DPSE * Workforce	12	-40.801	107.799	1.776	0.129	ALL
**DPSEA**	Linear Trend ~ Vaccination + Baseline + DPSE * Workforce	12	-42.786	111.768	5.745	0.018	ALL
**DPSEA**	Linear Trend ~ 1	3	-71.821	149.801	43.778	0.000	ALL
**DPSEA.noDr**	Linear Trend ~ DPSE + *General*	7	-52.507	119.614	0.000	0.420	ALL
**DPSEA.noDr**	Linear Trend ~ DPSE + *Action*	7	-53.329	121.256	1.643	0.185	ALL
**DPSEA.noDr**	Linear Trend ~ DPSE	6	-54.974	122.394	2.781	0.105	ALL
**DPSEA.noDr**	Linear Trend ~ DPSE + *Monitoring and Surveillance*	7	-54.244	123.086	3.473	0.074	ALL
**DPSEA.noDr**	Linear Trend ~ DPSE + *General* + Baseline	8	-54.337	125.448	5.834	0.023	ALL
**DPSEA.noDr**	Linear Trend ~ 1	3	-78.520	163.165	43.551	0.000	ALL
**aP.noDr**	Linear Trend ~ DPSE + *General*	7	-54.390	123.410	0.000	0.394	ALL
**aP.noDr**	Linear Trend ~ DPSE + *Action*	7	-55.026	124.682	1.272	0.209	ALL
**aP.noDr**	Linear Trend ~ DPSE	6	-56.727	125.924	2.514	0.112	ALL
**aP.noDr**	Linear Trend ~ DPSE + *Monitoring and Surveillance*	7	-55.771	126.172	2.762	0.099	ALL
**aP.noDr**	Linear Trend ~ DPSE + *General* + *Action*	8	-56.214	129.242	5.832	0.021	ALL
**aP.noDr**	Linear Trend ~ 1	3	-78.269	162.670	39.259	0.000	ALL
**aS.noDr**	Linear Trend ~ DPSE + *General*	7	-44.454	103.720	0.000	0.345	ALL
**aS.noDr**	Linear Trend ~ DPSE	6	-45.663	103.929	0.209	0.311	ALL
**aS.noDr**	Linear Trend ~ DPSE + *Action*	7	-45.650	106.112	2.392	0.104	ALL

**Model Name**	**Variables**	**df**	**logLik**	**AICc**	**delta**	**weight**	**income**
**aS.noDr**	Linear Trend ~ DPSE + *Monitoring and Surveillance*	7	-46.370	107.551	3.831	0.051	ALL
**aS.noDr**	Linear Trend ~ *Awareness and Education* + DPSE + *General*	8	-45.499	108.050	4.330	0.040	ALL
**aS.noDr**	Linear Trend ~ 1	3	-56.134	118.437	14.717	0.000	ALL
**aE.noDr**	Linear Trend ~ DPSE	6	-42.937	98.546	0.000	0.473	ALL
**aE.noDr**	Linear Trend ~ DPSE + *General*	7	-42.679	100.260	1.715	0.201	ALL
**aE.noDr**	Linear Trend ~ DPSE + *Action*	7	-43.425	101.754	3.208	0.095	ALL
**aE.noDr**	Linear Trend ~ DPSE + *Monitoring and Surveillance*	7	-43.622	102.147	3.601	0.078	ALL
**aE.noDr**	Linear Trend ~ *Awareness and Education* + DPSE	7	-44.668	104.240	5.694	0.027	ALL
**aE.noDr**	Linear Trend ~ 1	3	-50.749	107.686	9.140	0.005	ALL
**aP**	Linear Trend ~ DPSE * Workforce	10	-46.859	115.300	0.000	0.223	ALL
**aP**	Linear Trend ~ DPSE	6	-51.456	115.500	0.200	0.201	ALL
**aP**	Linear Trend ~ DPSE + *General*	7	-50.387	115.562	0.262	0.195	ALL
**aP**	Linear Trend ~ DPSE + *Action*	7	-51.183	117.154	1.854	0.088	ALL
**aP**	Linear Trend ~ DPSE + *Monitoring and Surveillance*	7	-52.167	119.122	3.822	0.033	ALL
**aP**	Linear Trend ~ 1	3	-71.261	148.686	33.386	0.000	ALL
**aS**	Linear Trend ~ DPSE	6	-43.015	98.853	0.000	0.296	ALL
**aS**	Linear Trend ~ DPSE * Sanitation	10	-38.625	99.495	0.641	0.215	ALL
**aS**	Linear Trend ~ DPSE + *General*	7	-42.719	100.547	1.694	0.127	ALL
**aS**	Linear Trend ~ DPSE + *Action*	7	-43.710	102.529	3.676	0.047	ALL
**aS**	Linear Trend ~ DPSE * Workforce	10	-40.240	102.725	3.871	0.043	ALL
**aS**	Linear Trend ~ 1	3	-50.850	107.928	9.075	0.003	ALL
**aE**	Linear Trend ~ DPSE	6	-39.877	92.667	0.000	0.313	ALL
**aE**	Linear Trend ~ DPSE * Workforce	10	-35.385	93.271	0.603	0.231	ALL
**aE**	Linear Trend ~ DPSE * Sanitation	10	-36.625	95.750	3.083	0.067	ALL
**aE**	Linear Trend ~ DPSE + *General*	7	-40.344	95.919	3.252	0.061	ALL
**aE**	Linear Trend ~ DPSE + *Action*	7	-40.895	97.021	4.354	0.035	ALL
**aE**	Linear Trend ~ 1	3	-45.471	97.195	4.528	0.032	ALL
**Dr**	Linear Trend ~ Baseline	5	-9.833	29.890	0.000	0.790	ALL
**Dr**	Linear Trend ~ *Action* + Baseline	6	-11.499	35.312	5.423	0.052	ALL
**Dr**	Linear Trend ~ *General* + Baseline	6	-11.615	35.546	5.656	0.047	ALL
**Dr**	Linear Trend ~ *Awareness and Education* + Baseline	6	-12.048	36.411	6.521	0.030	ALL
**Dr**	Linear Trend ~ income + Baseline	6	-12.144	36.604	6.714	0.028	ALL
**Dr**	Linear Trend ~ 1	4	-16.607	41.362	11.473	0.003	ALL
**P**	Linear Trend ~ *Monitoring and Surveillance*	5	-125.120	260.614	0.000	0.134	ALL
**P**	Linear Trend ~ Workforce	5	-125.162	260.699	0.085	0.128	ALL
**P**	Linear Trend ~ *Action*	5	-125.799	261.973	1.359	0.068	ALL
**P**	Linear Trend ~ 1	4	-126.877	262.002	1.388	0.067	ALL
**P**	Linear Trend ~ income	5	-125.877	262.130	1.515	0.063	ALL
**P**	Linear Trend ~ 1	4	-126.877	262.002	1.388	0.067	ALL
**S**	Linear Trend ~ income + Baseline	6	-53.321	120.228	0.000	0.083	ALL

Model Name	Variables	df	logLik	AICc	delta	weight	income
**S**	Linear Trend ~ *Monitoring and Surveillance* + Baseline	6	-53.491	120.566	0.338	0.070	ALL
**S**	Linear Trend ~ Baseline	5	-54.894	120.900	0.672	0.059	ALL
**S**	Linear Trend ~ Infection + Mean Temperature + Baseline	7	-52.563	121.279	1.052	0.049	ALL
**S**	Linear Trend ~ Mean Temperature + Baseline	6	-53.871	121.326	1.099	0.048	ALL
**S**	Linear Trend ~ 1	4	-57.515	123.757	3.530	0.014	ALL
**E**	Linear Trend ~ income + Mean Temperature + *Action*	5	7.656	0.142	0.000	0.368	ALL
**E**	Linear Trend ~ *General* + income + Mean Temperature	5	6.901	1.652	1.510	0.173	ALL
**E**	Linear Trend ~ *Awareness and Education* + income + Mean Temperature	5	6.054	3.346	3.205	0.074	ALL
**E**	Linear Trend ~ income + Workforce + Baseline	5	5.353	4.749	4.607	0.037	ALL
**E**	Linear Trend ~ income + Mean Temperature + *Monitoring and Surveillance*	5	5.285	4.884	4.742	0.034	ALL
**E**	Linear Trend ~ 1	2	-7.159	19.174	19.032	0.000	ALL
**DPS**	Linear Trend ~ DPSE	5	-42.065	94.584	0.000	0.243	ALL
**DPS**	Linear Trend ~ DPSE + *General*	6	-41.273	95.188	0.604	0.179	ALL
**DPS**	Linear Trend ~ DPSE + *Action*	6	-42.150	96.941	2.358	0.075	ALL
**DPS**	Linear Trend ~ DPSE * Workforce	8	-40.105	97.327	2.743	0.062	ALL
**DPS**	Linear Trend ~ DPSE + *Action* + Baseline	7	-41.402	97.665	3.081	0.052	ALL
**DPS**	Linear Trend ~ 1	3	-64.124	134.427	39.843	0.000	ALL
**PSE**	Linear Trend ~ *Monitoring and Surveillance*	4	-47.422	103.283	0.000	0.204	ALL
**PSE**	Linear Trend ~ *Action*	4	-48.224	104.888	1.605	0.092	ALL
**PSE**	Linear Trend ~ income	4	-48.881	106.202	2.919	0.047	ALL
**PSE**	Linear Trend ~ 1	3	-50.434	107.129	3.846	0.030	ALL
**PSE**	Linear Trend ~ *General*	4	-49.358	107.155	3.872	0.029	ALL
**PSE**	Linear Trend ~ 1	3	-50.434	107.129	3.846	0.030	ALL
**DP**	Linear Trend ~ DPSE * Workforce	6	-21.956	56.713	0.000	0.258	ALL
**DP**	Linear Trend ~ DPSE * *Monitoring and Surveillance*	6	-22.670	58.140	1.427	0.126	ALL
**DP**	Linear Trend ~ DPSE	4	-24.923	58.220	1.507	0.121	ALL
**DP**	Linear Trend ~ DPSE + *Monitoring and Surveillance* + Baseline	6	-22.782	58.364	1.651	0.113	ALL
**DP**	Linear Trend ~ DPSE + *Action* + Baseline	6	-23.561	59.923	3.210	0.052	ALL
**DP**	Linear Trend ~ 1	3	-50.092	106.405	49.693	0.000	ALL
**PS**	Linear Trend ~ Baseline * income	6	-36.503	86.172	0.000	0.425	ALL
**PS**	Linear Trend ~ *Monitoring and Surveillance*	4	-40.499	89.538	3.366	0.079	ALL
**PS**	Linear Trend ~ income	4	-41.170	90.881	4.709	0.040	ALL
**PS**	Linear Trend ~ Baseline + income	5	-40.237	91.297	5.124	0.033	ALL
**PS**	Linear Trend ~ *Monitoring and Surveillance* + Baseline	5	-40.307	91.435	5.263	0.031	ALL
**PS**	Linear Trend ~ 1	3	-44.120	94.560	8.387	0.006	ALL

Model Name	Variables	df	logLik	AICc	delta	weight	income
**SE**	Linear Trend ~ Infection + Mean Temperature + Workforce	6	-17.852	50.249	0.000	0.083	ALL
**SE**	Linear Trend ~ Sanitation * income	6	-18.165	50.875	0.626	0.061	ALL
**SE**	Linear Trend ~ 1	3	-22.129	50.925	0.676	0.059	ALL
**SE**	Linear Trend ~ Baseline * income	6	-18.258	51.061	0.812	0.055	ALL
**SE**	Linear Trend ~ Infection + Workforce	5	-19.747	51.258	1.009	0.050	ALL
**SE**	Linear Trend ~ 1	3	-22.129	50.925	0.676	0.059	ALL
**DPSEA**	Linear Trend ~ DPSE + *Monitoring and Surveillance*	7	-20.925	57.138	0.000	0.083	HIC
**DPSEA**	Linear Trend ~ *General* + Mean Temperature + DPSE * Workforce	12	-14.971	57.746	0.608	0.061	HIC
**DPSEA**	Linear Trend ~ *Monitoring and Surveillance* + DPSE * Workforce	11	-16.400	57.981	0.842	0.054	HIC
**DPSEA**	Linear Trend ~ DPSE	6	-22.563	58.081	0.942	0.052	HIC
**DPSEA**	Linear Trend ~ Mean Temperature + *Action* + DPSE * Workforce	12	-15.399	58.604	1.466	0.040	HIC
**DPSEA**	Linear Trend ~ 1	3	-28.844	63.951	6.813	0.003	HIC
**DPSEA.noDr**	Linear Trend ~ DPSE + *Monitoring and Surveillance*	7	-18.484	51.958	0.000	0.479	HIC
**DPSEA.noDr**	Linear Trend ~ DPSE + *General*	7	-20.233	55.458	3.499	0.083	HIC
**DPSEA.noDr**	Linear Trend ~ DPSE + *Action*	7	-20.444	55.879	3.921	0.067	HIC
**DPSEA.noDr**	Linear Trend ~ DPSE	6	-21.759	56.255	4.297	0.056	HIC
**DPSEA.noDr**	Linear Trend ~ DPSE * *Monitoring and Surveillance*	10	-17.210	56.419	4.461	0.051	HIC
**DPSEA.noDr**	Linear Trend ~ 1	3	-31.466	69.138	17.179	0.000	HIC
**aP.noDr**	Linear Trend ~ DPSE + *Monitoring and Surveillance*	7	-20.371	55.788	0.000	0.478	HIC
**aP.noDr**	Linear Trend ~ DPSE + *General*	7	-22.203	59.453	3.665	0.077	HIC
**aP.noDr**	Linear Trend ~ DPSE	6	-23.458	59.695	3.906	0.068	HIC
**aP.noDr**	Linear Trend ~ DPSE + *Action*	7	-22.366	59.779	3.991	0.065	HIC
**aP.noDr**	Linear Trend ~ DPSE + *General* + Mean Temperature	8	-21.403	60.164	4.376	0.054	HIC
**aP.noDr**	Linear Trend ~ 1	3	-32.479	71.174	15.386	0.000	HIC
**aS.noDr**	Linear Trend ~ DPSE	6	-16.013	44.850	0.000	0.295	HIC
**aS.noDr**	Linear Trend ~ DPSE + *General*	7	-15.157	45.423	0.573	0.222	HIC
**aS.noDr**	Linear Trend ~ DPSE + *Monitoring and Surveillance*	7	-15.597	46.304	1.454	0.143	HIC
**aS.noDr**	Linear Trend ~ DPSE + *Action*	7	-15.988	47.085	2.235	0.097	HIC
**aS.noDr**	Linear Trend ~ DPSE + Baseline	7	-16.755	48.619	3.769	0.045	HIC
**aS.noDr**	Linear Trend ~ 1	3	-23.229	52.686	7.836	0.006	HIC
**aE.noDr**	Linear Trend ~ DPSE	6	-17.860	48.596	0.000	0.322	HIC
**aE.noDr**	Linear Trend ~ DPSE + *General*	7	-17.271	49.722	1.126	0.184	HIC
**aE.noDr**	Linear Trend ~ DPSE + *Monitoring and Surveillance*	7	-17.493	50.165	1.569	0.147	HIC
**aE.noDr**	Linear Trend ~ DPSE + *Action*	7	-18.018	51.216	2.620	0.087	HIC
**aE.noDr**	Linear Trend ~ DPSE + Baseline	7	-18.792	52.762	4.166	0.040	HIC
**aE.noDr**	Linear Trend ~ 1	3	-24.501	55.245	6.649	0.012	HIC
**aP**	Linear Trend ~ DPSE * Workforce	10	-18.012	58.774	0.000	0.190	HIC
**aP**	Linear Trend ~ DPSE + *Monitoring and Surveillance*	7	-21.976	59.300	0.526	0.146	HIC
**aP**	Linear Trend ~ DPSE	6	-23.598	60.196	1.422	0.093	HIC

Model Name	Variables	df	logLik	AICc	delta	weight	income
**aP**	Linear Trend ~ DPSE + *Monitoring and Surveillance* + Workforce	8	-21.730	61.217	2.443	0.056	HIC
**aP**	Linear Trend ~ DPSE + Infection + *Monitoring and Surveillance*	8	-21.777	61.310	2.536	0.053	HIC
**aP**	Linear Trend ~ 1	3	-29.328	64.933	6.159	0.009	HIC
**aS**	Linear Trend ~ DPSE	6	-17.385	47.876	0.000	0.120	HIC
**aS**	Linear Trend ~ 1	3	-21.007	48.319	0.443	0.096	HIC
**aS**	Linear Trend ~ DPSE * Infection	10	-13.263	49.581	1.705	0.051	HIC
**aS**	Linear Trend ~ DPSE + Infection	7	-17.148	49.790	1.914	0.046	HIC
**aS**	Linear Trend ~ Infection	4	-20.769	50.051	2.175	0.040	HIC
**aS**	Linear Trend ~ 1	3	-21.007	48.319	0.443	0.096	HIC
**aE**	Linear Trend ~ 1	3	-21.685	49.690	0.000	0.100	HIC
**aE**	Linear Trend ~ DPSE	6	-18.520	50.207	0.517	0.077	HIC
**aE**	Linear Trend ~ Workforce	4	-21.122	50.784	1.094	0.058	HIC
**aE**	Linear Trend ~ Infection	4	-21.388	51.317	1.627	0.044	HIC
**aE**	Linear Trend ~ *Monitoring and Surveillance* + Workforce	5	-20.292	51.406	1.716	0.043	HIC
**aE**	Linear Trend ~ 1	3	-21.685	49.690	0.000	0.100	HIC
**Dr**	Linear Trend ~ Baseline	5	26.283	-42.114	0.000	0.587	HIC
**Dr**	Linear Trend ~ *Action* + Baseline	6	26.156	-39.676	2.438	0.174	HIC
**Dr**	Linear Trend ~ *Monitoring and Surveillance* + Baseline	6	25.471	-38.305	3.809	0.087	HIC
**Dr**	Linear Trend ~ 1	4	22.677	-37.056	5.058	0.047	HIC
**Dr**	Linear Trend ~ *Awareness and Education* + Baseline	6	24.562	-36.488	5.625	0.035	HIC
**Dr**	Linear Trend ~ 1	4	22.677	-37.056	5.058	0.047	HIC
**P**	Linear Trend ~ *Monitoring and Surveillance*	5	-53.675	118.119	0.000	0.154	HIC
**P**	Linear Trend ~ Mean Temperature	5	-54.189	119.148	1.029	0.092	HIC
**P**	Linear Trend ~ Infection + *Monitoring and Surveillance*	6	-53.038	119.166	1.047	0.091	HIC
**P**	Linear Trend ~ Infection + Workforce	6	-53.714	120.518	2.399	0.046	HIC
**P**	Linear Trend ~ Workforce	5	-54.975	120.718	2.599	0.042	HIC
**P**	Linear Trend ~ 1	4	-57.569	123.645	5.526	0.010	HIC
**S**	Linear Trend ~ Mean Temperature + Workforce + Baseline	7	-48.134	112.601	0.000	0.133	HIC
**S**	Linear Trend ~ Infection + Baseline	6	-49.926	113.566	0.965	0.082	HIC
**S**	Linear Trend ~ Baseline	5	-51.259	113.718	1.117	0.076	HIC
**S**	Linear Trend ~ Infection + *Monitoring and Surveillance* + Baseline	7	-49.055	114.444	1.843	0.053	HIC
**S**	Linear Trend ~ Mean Temperature + Baseline	6	-50.514	114.742	2.141	0.046	HIC
**S**	Linear Trend ~ 1	4	-53.550	115.884	3.284	0.026	HIC
**E**	Linear Trend ~ Mean Temperature + *Action* + Workforce	5	11.959	-7.919	0.000	0.650	HIC
**E**	Linear Trend ~ Mean Temperature + *Action* + Baseline	5	9.132	-2.265	5.654	0.038	HIC
**E**	Linear Trend ~ Mean Temperature + *Action*	4	6.721	-1.806	6.113	0.031	HIC
**E**	Linear Trend ~ *General* + Mean Temperature + Workforce	5	8.753	-1.506	6.413	0.026	HIC

**Model Name**	**Variables**	**df**	**logLik**	**AICc**	**delta**	**weight**	**income**
**E**	Linear Trend ~ Mean Temperature + *Monitoring and Surveillance* + Workforce	5	8.440	-0.880	7.039	0.019	HIC
**E**	Linear Trend ~ 1	2	-1.235	7.394	15.312	0.000	HIC
**DPS**	Linear Trend ~ DPSE * Workforce	8	-16.709	51.475	0.000	0.207	HIC
**DPS**	Linear Trend ~ DPSE	5	-20.646	52.115	0.640	0.150	HIC
**DPS**	Linear Trend ~ DPSE + *Monitoring and Surveillance*	6	-19.923	53.013	1.538	0.096	HIC
**DPS**	Linear Trend ~ DPSE + Infection	6	-20.759	54.685	3.210	0.042	HIC
**DPS**	Linear Trend ~ DPSE + Infection + *Monitoring and Surveillance*	7	-19.686	54.950	3.475	0.036	HIC
**DPS**	Linear Trend ~ 1	3	-24.602	55.524	4.049	0.027	HIC
**PSE**	Linear Trend ~ Mean Temperature	4	-22.094	52.855	0.000	0.093	HIC
**PSE**	Linear Trend ~ *Monitoring and Surveillance*	4	-22.286	53.239	0.384	0.077	HIC
**PSE**	Linear Trend ~ Mean Temperature + *Action* + Workforce	6	-19.982	53.412	0.557	0.071	HIC
**PSE**	Linear Trend ~ *General* + Mean Temperature + Workforce	6	-19.995	53.439	0.584	0.070	HIC
**PSE**	Linear Trend ~ Infection + *Monitoring and Surveillance*	5	-21.363	53.743	0.888	0.060	HIC
**PSE**	Linear Trend ~ 1	3	-25.166	56.725	3.870	0.013	HIC
**DP**	Linear Trend ~ DPSE * *Monitoring and Surveillance*	6	4.630	4.454	0.000	0.505	HIC
**DP**	Linear Trend ~ DPSE + *Monitoring and Surveillance*	5	2.065	7.070	2.616	0.137	HIC
**DP**	Linear Trend ~ DPSE * Mean Temperature	6	3.095	7.525	3.072	0.109	HIC
**DP**	Linear Trend ~ DPSE * Workforce	6	2.718	8.278	3.825	0.075	HIC
**DP**	Linear Trend ~ DPSE + Infection + *Monitoring and Surveillance*	6	1.806	10.103	5.649	0.030	HIC
**DP**	Linear Trend ~ 1	3	-9.326	25.114	20.661	0.000	HIC
**PS**	Linear Trend ~ Mean Temperature	4	-20.747	50.404	0.000	0.105	HIC
**PS**	Linear Trend ~ *Monitoring and Surveillance*	4	-20.837	50.583	0.179	0.096	HIC
**PS**	Linear Trend ~ Infection + *Monitoring and Surveillance*	5	-19.859	51.113	0.709	0.073	HIC
**PS**	Linear Trend ~ Infection + Mean Temperature	5	-20.441	52.276	1.873	0.041	HIC
**PS**	Linear Trend ~ 1	3	-22.953	52.439	2.035	0.038	HIC
**PS**	Linear Trend ~ 1	3	-22.953	52.439	2.035	0.038	HIC
**SE**	Linear Trend ~ Workforce + Baseline	5	-15.131	42.198	0.000	0.054	HIC
**SE**	Linear Trend ~ Mean Temperature + Workforce	5	-15.294	42.524	0.326	0.046	HIC
**SE**	Linear Trend ~ 1	3	-17.998	42.723	0.526	0.042	HIC
**SE**	Linear Trend ~ DPSE * Infection	6	-14.010	42.820	0.622	0.040	HIC
**SE**	Linear Trend ~ Infection + Mean Temperature + Workforce	6	-14.137	43.075	0.877	0.035	HIC
**SE**	Linear Trend ~ 1	3	-17.998	42.723	0.526	0.042	HIC
**DPSEA**	Linear Trend ~ DPSE * Baseline	9	-6.856	35.313	0.000	0.289	LMIC
**DPSEA**	Linear Trend ~ DPSE + Baseline	7	-10.081	36.315	1.002	0.175	LMIC

Model Name	Variables	df	logLik	AICc	delta	weight	income
**DPSEA**	Linear Trend ~ DPSE + Workforce + Baseline	8	-9.803	38.430	3.117	0.061	LMIC
**DPSEA**	Linear Trend ~ Workforce + Baseline + DPSE * Sanitation	11	-5.594	38.687	3.374	0.053	LMIC
**DPSEA**	Linear Trend ~ Workforce + DPSE * Baseline	10	-7.360	39.210	3.897	0.041	LMIC
**DPSEA**	Linear Trend ~ 1	3	-39.742	85.913	50.600	0.000	LMIC
**DPSEA.noDr**	Linear Trend ~ DPSE * Baseline	10	-14.246	51.984	0.000	0.700	LMIC
**DPSEA.noDr**	Linear Trend ~ *Monitoring and Surveillance* + DPSE * Baseline	11	-15.220	56.698	4.713	0.066	LMIC
**DPSEA.noDr**	Linear Trend ~ Baseline + DPSE * *Monitoring and Surveillance*	11	-15.567	57.392	5.408	0.047	LMIC
**DPSEA.noDr**	Linear Trend ~ *Action* + DPSE * Baseline	11	-15.592	57.442	5.458	0.046	LMIC
**DPSEA.noDr**	Linear Trend ~ *General* + DPSE * Baseline	11	-16.172	58.602	6.618	0.026	LMIC
**DPSEA.noDr**	Linear Trend ~ 1	3	-43.699	93.740	41.756	0.000	LMIC
**aP.noDr**	Linear Trend ~ DPSE * Baseline	10	-15.008	53.683	0.000	0.945	LMIC
**aP.noDr**	Linear Trend ~ DPSE + Baseline	7	-22.175	60.127	6.444	0.038	LMIC
**aP.noDr**	Linear Trend ~ DPSE + *Action* + Baseline	8	-22.727	63.776	10.093	0.006	LMIC
**aP.noDr**	Linear Trend ~ DPSE + *Monitoring and Surveillance* + Baseline	8	-23.342	65.006	11.323	0.003	LMIC
**aP.noDr**	Linear Trend ~ DPSE + *General* + Baseline	8	-23.569	65.460	11.777	0.003	LMIC
**aP.noDr**	Linear Trend ~ 1	3	-42.820	91.999	38.316	0.000	LMIC
**aS.noDr**	Linear Trend ~ DPSE * Baseline	10	-10.104	48.669	0.000	0.755	LMIC
**aS.noDr**	Linear Trend ~ DPSE	6	-18.966	52.732	4.063	0.099	LMIC
**aS.noDr**	Linear Trend ~ DPSE + Baseline	7	-17.745	53.353	4.684	0.073	LMIC
**aS.noDr**	Linear Trend ~ DPSE + *General*	7	-19.883	57.628	8.959	0.009	LMIC
**aS.noDr**	Linear Trend ~ *Awareness and Education* + DPSE	7	-19.915	57.692	9.023	0.008	LMIC
**aS.noDr**	Linear Trend ~ 1	3	-26.605	59.936	11.267	0.003	LMIC
**aE.noDr**	Linear Trend ~ DPSE	6	-16.354	48.526	0.000	0.361	LMIC
**aE.noDr**	Linear Trend ~ 1	3	-21.386	49.733	1.206	0.198	LMIC
**aE.noDr**	Linear Trend ~ DPSE + Baseline	7	-16.324	51.981	3.454	0.064	LMIC
**aE.noDr**	Linear Trend ~ Baseline	4	-21.484	52.635	4.109	0.046	LMIC
**aE.noDr**	Linear Trend ~ DPSE + *Monitoring and Surveillance*	7	-17.137	53.608	5.082	0.028	LMIC
**aE.noDr**	Linear Trend ~ 1	3	-21.386	49.733	1.206	0.198	LMIC
**aP**	Linear Trend ~ DPSE * Baseline	9	-7.245	36.163	0.000	0.450	LMIC
**aP**	Linear Trend ~ DPSE + Baseline	7	-10.325	36.847	0.683	0.320	LMIC
**aP**	Linear Trend ~ DPSE + Workforce + Baseline	8	-10.135	39.149	2.986	0.101	LMIC
**aP**	Linear Trend ~ DPSE + *Action* + Baseline	8	-11.538	41.957	5.793	0.025	LMIC
**aP**	Linear Trend ~ DPSE + *General* + Baseline	8	-11.686	42.253	6.090	0.021	LMIC
**aP**	Linear Trend ~ 1	3	-39.203	84.842	48.679	0.000	LMIC
**aS**	Linear Trend ~ DPSE * Baseline	8	2.740	18.991	0.000	0.329	LMIC
**aS**	Linear Trend ~ DPSE + Baseline	6	-2.181	20.782	1.791	0.134	LMIC
**aS**	Linear Trend ~ DPSE + Workforce + Baseline	7	-0.626	21.474	2.483	0.095	LMIC

Model Name	Variables	df	logLik	AICc	delta	weight	income
**aS**	Linear Trend ~ DPSE + *General* + Baseline	7	-0.722	21.666	2.675	0.086	LMIC
**aS**	Linear Trend ~ DPSE + *Action* + Baseline	7	-1.081	22.384	3.392	0.060	LMIC
**aS**	Linear Trend ~ 1	2	-21.148	46.819	27.827	0.000	LMIC
**aE**	Linear Trend ~ DPSE + Baseline	6	-1.744	21.950	0.000	0.231	LMIC
**aE**	Linear Trend ~ DPSE + Workforce + Baseline	7	0.435	22.463	0.512	0.179	LMIC
**aE**	Linear Trend ~ *Awareness and Education* + DPSE + Baseline	7	0.129	23.076	1.126	0.131	LMIC
**aE**	Linear Trend ~ DPSE * Baseline	7	-0.105	23.542	1.592	0.104	LMIC
**aE**	Linear Trend ~ DPSE + Animal Production + Baseline	7	-0.475	24.283	2.333	0.072	LMIC
**aE**	Linear Trend ~ 1	2	-16.632	37.969	16.019	0.000	LMIC
**Dr**	Linear Trend ~ 1	4	-28.779	65.867	0.000	0.547	LMIC
**Dr**	Linear Trend ~ Baseline	5	-28.881	68.227	2.360	0.168	LMIC
**Dr**	Linear Trend ~ *General*	5	-29.749	69.962	4.096	0.071	LMIC
**Dr**	Linear Trend ~ *Action*	5	-30.312	71.089	5.222	0.040	LMIC
**Dr**	Linear Trend ~ *Awareness and Education*	5	-30.876	72.216	6.350	0.023	LMIC
**Dr**	Linear Trend ~ 1	4	-28.779	65.867	0.000	0.547	LMIC
**P**	Linear Trend ~ 1	4	-66.595	141.710	0.000	0.342	LMIC
**P**	Linear Trend ~ Sanitation	5	-66.919	144.627	2.917	0.080	LMIC
**P**	Linear Trend ~ *Awareness and Education*	5	-67.456	145.701	3.990	0.047	LMIC
**P**	Linear Trend ~ *General*	5	-67.559	145.908	4.198	0.042	LMIC
**P**	Linear Trend ~ *Action*	5	-67.564	145.918	4.208	0.042	LMIC
**P**	Linear Trend ~ 1	4	-66.595	141.710	0.000	0.342	LMIC
**DPS**	Linear Trend ~ DPSE * Baseline	8	-6.856	32.593	0.000	0.503	LMIC
**DPS**	Linear Trend ~ DPSE + Baseline	6	-10.081	33.777	1.184	0.278	LMIC
**DPS**	Linear Trend ~ DPSE + Workforce + Baseline	7	-9.803	35.803	3.210	0.101	LMIC
**DPS**	Linear Trend ~ DPSE + *Action* + Baseline	7	-11.222	38.641	6.048	0.024	LMIC
**DPS**	Linear Trend ~ DPSE + *General* + Baseline	7	-11.381	38.958	6.365	0.021	LMIC
**DPS**	Linear Trend ~ 1	3	-37.062	80.560	47.967	0.000	LMIC
**PSE**	Linear Trend ~ DPSE + Baseline	6	-11.667	38.834	0.000	0.533	LMIC
**PSE**	Linear Trend ~ DPSE * Baseline	7	-11.137	41.143	2.309	0.168	LMIC
**PSE**	Linear Trend ~ DPSE + Sanitation + Baseline	7	-12.188	43.246	4.413	0.059	LMIC
**PSE**	Linear Trend ~ *Awareness and Education* + DPSE + Baseline	7	-12.506	43.881	5.047	0.043	LMIC
**PSE**	Linear Trend ~ DPSE + Workforce + Baseline	7	-12.756	44.381	5.547	0.033	LMIC
**PSE**	Linear Trend ~ 1	3	-21.942	50.774	11.940	0.001	LMIC
**DP**	Linear Trend ~ DPSE * Baseline	6	-7.860	29.435	0.000	0.337	LMIC
**DP**	Linear Trend ~ DPSE + Baseline	5	-9.182	29.565	0.130	0.316	LMIC
**DP**	Linear Trend ~ DPSE + Workforce + Baseline	6	-8.308	30.331	0.896	0.215	LMIC
**DP**	Linear Trend ~ DPSE + *Action* + Baseline	6	-10.561	34.837	5.402	0.023	LMIC

Model Name	Variables	df	logLik	AICc	delta	weight	income
**DP**	Linear Trend ~ DPSE + Sanitation + Baseline	6	-10.593	34.900	5.465	0.022	LMIC
**DP**	Linear Trend ~ 1	3	-35.811	78.084	48.649	0.000	LMIC
**PS**	Linear Trend ~ Baseline	4	-9.237	28.075	0.000	0.518	LMIC
**PS**	Linear Trend ~ Sanitation + Baseline	5	-9.767	32.034	3.959	0.072	LMIC
**PS**	Linear Trend ~ *Awareness and Education* + Baseline	5	-10.081	32.662	4.587	0.052	LMIC
**PS**	Linear Trend ~ Workforce + Baseline	5	-10.307	33.115	5.040	0.042	LMIC
**PS**	Linear Trend ~ Infection + Baseline	5	-10.345	33.190	5.116	0.040	LMIC
**PS**	Linear Trend ~ 1	3	-17.831	42.585	14.511	0.000	LMIC
**DPSEA**	Categorical Trend ~ DPSE * Gini + DPSE * Mean Temperature	13	-33.291	95.163	0.000	0.600	ALL
**DPSEA**	Categorical Trend ~ DPSE * Mean Temperature + DPSE * Workforce	13	-36.107	100.795	5.633	0.036	ALL
**DPSEA**	Categorical Trend ~ Workforce + DPSE * Mean Temperature	10	-40.197	101.921	6.759	0.020	ALL
**DPSEA**	Categorical Trend ~ *General* + Workforce + DPSE * Mean Temperature	11	-39.562	102.969	7.807	0.012	ALL
**DPSEA**	Categorical Trend ~ GDP + Workforce + DPSE * Mean Temperature	11	-39.655	103.155	7.993	0.011	ALL
**DPSEA**	Categorical Trend ~ 1	2	-107.357	218.793	123.631	0.000	ALL
**DPSEA.noDr**	Categorical Trend ~ Animal Production + *Action* + DPSE * Mean Temperature	11	-56.943	137.328	0.000	0.514	ALL
**DPSEA.noDr**	Categorical Trend ~ *General* + Animal Production + DPSE * Mean Temperature	11	-57.691	138.824	1.496	0.243	ALL
**DPSEA.noDr**	Categorical Trend ~ *Awareness and Education* + Animal Production + DPSE * Mean Temperature	11	-59.994	143.431	6.103	0.024	ALL
**DPSEA.noDr**	Categorical Trend ~ *General* + DPSE * Mean Temperature	10	-61.421	144.037	6.709	0.018	ALL
**DPSEA.noDr**	Categorical Trend ~ *General* + Baseline + DPSE * Mean Temperature	11	-60.511	144.465	7.137	0.014	ALL
**DPSEA.noDr**	Categorical Trend ~ 1	2	-135.161	274.385	137.057	0.000	ALL
**aP.noDr**	Categorical Trend ~ DPSE + *General* + Animal Production	7	-64.701	144.032	0.000	0.218	ALL
**aP.noDr**	Categorical Trend ~ DPSE * Mean Temperature	9	-62.586	144.195	0.163	0.200	ALL
**aP.noDr**	Categorical Trend ~ DPSE * *Monitoring and Surveillance*	9	-63.573	146.169	2.137	0.075	ALL
**aP.noDr**	Categorical Trend ~ DPSE + *General*	6	-66.951	146.371	2.339	0.068	ALL
**aP.noDr**	Categorical Trend ~ DPSE + Animal Production + *Action*	7	-66.036	146.701	2.669	0.057	ALL
**aP.noDr**	Categorical Trend ~ 1	2	-128.882	261.830	117.798	0.000	ALL

**Model Name**	**Variables**	**df**	**logLik**	**AICc**	**delta**	**weight**	**income**
**aS.noDr**	Categorical Trend ~ DPSE + *General* + Animal Production	7	-51.992	118.796	0.000	0.397	ALL
**aS.noDr**	Categorical Trend ~ DPSE + Animal Production + *Action*	7	-52.756	120.324	1.528	0.185	ALL
**aS.noDr**	Categorical Trend ~ *Awareness and Education* + DPSE + Animal Production	7	-53.625	122.061	3.265	0.078	ALL
**aS.noDr**	Categorical Trend ~ DPSE + *General*	6	-55.090	122.784	3.988	0.054	ALL
**aS.noDr**	Categorical Trend ~ *Awareness and Education* + DPSE + *General*	7	-54.748	124.308	5.512	0.025	ALL
**aS.noDr**	Categorical Trend ~ 1	2	-100.980	206.044	87.248	0.000	ALL
**aE.noDr**	Categorical Trend ~ DPSE + *General* + Animal Production	7	-48.671	112.246	0.000	0.292	ALL
**aE.noDr**	Categorical Trend ~ DPSE + Animal Production + *Action*	7	-49.031	112.965	0.719	0.204	ALL
**aE.noDr**	Categorical Trend ~ *Awareness and Education* + DPSE + Animal Production	7	-49.630	114.163	1.917	0.112	ALL
**aE.noDr**	Categorical Trend ~ DPSE + *General*	6	-51.549	115.769	3.523	0.050	ALL
**aE.noDr**	Categorical Trend ~ *Awareness and Education* + DPSE + *General*	7	-51.208	117.319	5.073	0.023	ALL
**aE.noDr**	Categorical Trend ~ 1	2	-91.080	186.253	74.007	0.000	ALL
**aP**	Categorical Trend ~ DPSE * Mean Temperature	9	-34.041	87.367	0.000	0.997	ALL
**aP**	Categorical Trend ~ DPSE * Sanitation	9	-40.224	99.733	12.366	0.002	ALL
**aP**	Categorical Trend ~ DPSE * Workforce	9	-41.540	102.366	14.998	0.001	ALL
**aP**	Categorical Trend ~ DPSE * Gini	9	-41.746	102.778	15.410	0.000	ALL
**aP**	Categorical Trend ~ DPSE * Baseline	9	-42.658	104.601	17.234	0.000	ALL
**aP**	Categorical Trend ~ 1	2	-103.852	211.786	124.419	0.000	ALL
**aS**	Categorical Trend ~ DPSE + *Action* + Workforce	7	-36.487	88.083	0.000	0.219	ALL
**aS**	Categorical Trend ~ DPSE + *General* + Workforce	7	-36.683	88.475	0.393	0.180	ALL
**aS**	Categorical Trend ~ DPSE + *General* + Animal Production	7	-37.003	89.115	1.032	0.131	ALL
**aS**	Categorical Trend ~ DPSE + Animal Production + *Action*	7	-38.329	91.767	3.685	0.035	ALL
**aS**	Categorical Trend ~ DPSE + *General*	6	-39.615	92.053	3.971	0.030	ALL
**aS**	Categorical Trend ~ 1	2	-74.774	153.660	65.578	0.000	ALL
**aE**	Categorical Trend ~ DPSE * Mean Temperature	9	-24.729	69.480	0.000	0.989	ALL
**aE**	Categorical Trend ~ DPSE * Sanitation	9	-30.653	81.329	11.849	0.003	ALL
**aE**	Categorical Trend ~ DPSE + *Action* + Workforce	7	-33.254	81.738	12.259	0.002	ALL

**Model Name**	**Variables**	**df**	**logLik**	**AICc**	**delta**	**weight**	**income**
**aE**	Categorical Trend ~ DPSE + *General* + Workforce	7	-34.057	83.344	13.865	0.001	ALL
**aE**	Categorical Trend ~ DPSE + *General* + Animal Production	7	-34.167	83.565	14.085	0.001	ALL
**aE**	Categorical Trend ~ 1	2	-67.705	139.535	70.055	0.000	ALL
**Dr**	Categorical Trend ~ GDP + Baseline	4	-132.757	273.664	0.000	0.065	ALL
**Dr**	Categorical Trend ~ GDP + Mean Temperature + Baseline	5	-132.005	274.233	0.570	0.049	ALL
**Dr**	Categorical Trend ~ *General* * income	5	-132.019	274.262	0.598	0.048	ALL
**Dr**	Categorical Trend ~ GDP	3	-134.323	274.736	1.072	0.038	ALL
**Dr**	Categorical Trend ~ GDP + Mean Temperature	4	-133.386	274.921	1.257	0.034	ALL
**Dr**	Categorical Trend ~ 1	2	-142.735	289.515	15.851	0.000	ALL
**P**	Categorical Trend ~ *Monitoring and Surveillance* + Baseline	5	-70.903	152.182	0.000	0.081	ALL
**P**	Categorical Trend ~ Infection + *Monitoring and Surveillance* + Baseline	6	-70.199	152.925	0.744	0.056	ALL
**P**	Categorical Trend ~ *Action* + Baseline	5	-71.307	152.990	0.808	0.054	ALL
**P**	Categorical Trend ~ Mean Temperature + *Monitoring and Surveillance* + Baseline	6	-70.423	153.375	1.193	0.045	ALL
**P**	Categorical Trend ~ *Monitoring and Surveillance* + Workforce + Baseline	6	-70.578	153.684	1.503	0.038	ALL
**P**	Categorical Trend ~ 1	3	-83.958	174.063	21.882	0.000	ALL
**S**	Categorical Trend ~ GDP + *Monitoring and Surveillance* + Baseline	6	-27.018	67.621	0.000	0.087	ALL
**S**	Categorical Trend ~ *Monitoring and Surveillance* + Baseline	5	-28.903	68.916	1.295	0.046	ALL
**S**	Categorical Trend ~ Infection + *Monitoring and Surveillance* + Baseline	6	-27.896	69.377	1.756	0.036	ALL
**S**	Categorical Trend ~ *Monitoring and Surveillance* + Vaccination + Baseline	6	-27.916	69.417	1.796	0.035	ALL
**S**	Categorical Trend ~ *Awareness and Education* + Mean Temperature + Baseline	6	-28.090	69.764	2.143	0.030	ALL
**S**	Categorical Trend ~ 1	3	-41.555	89.538	21.917	0.000	ALL
**DPS**	Categorical Trend ~ DPSE * Mean Temperature	7	-38.065	90.992	0.000	0.097	ALL
**DPS**	Categorical Trend ~ DPSE	4	-42.536	93.373	2.381	0.030	ALL
**DPS**	Categorical Trend ~ DPSE + *General*	5	-41.507	93.470	2.478	0.028	ALL
**DPS**	Categorical Trend ~ DPSE + *Action*	5	-41.651	93.758	2.766	0.024	ALL
**DPS**	Categorical Trend ~ *Awareness and Education* + DPSE	5	-41.711	93.877	2.885	0.023	ALL

**Model Name**	**Variables**	**df**	**logLik**	**AICc**	**delta**	**weight**	**income**
**DPS**	Categorical Trend ~ 1	2	-95.524	195.137	104.145	0.000	ALL
**PSE**	Categorical Trend ~ DPSE * Sanitation	7	-25.576	66.424	0.000	0.686	ALL
**PSE**	Categorical Trend ~ DPSE * Workforce	7	-26.711	68.695	2.271	0.220	ALL
**PSE**	Categorical Trend ~ DPSE * Gini	7	-27.928	71.130	4.706	0.065	ALL
**PSE**	Categorical Trend ~ DPSE * Mean Temperature	7	-28.946	73.165	6.741	0.024	ALL
**PSE**	Categorical Trend ~ DPSE * Baseline	7	-31.982	79.237	12.813	0.001	ALL
**PSE**	Categorical Trend ~ 1	2	-49.352	102.832	36.408	0.000	ALL
**DP**	Categorical Trend ~ DPSE	3	-18.732	43.685	0.000	0.055	ALL
**DP**	Categorical Trend ~ DPSE + *General*	4	-18.561	45.497	1.811	0.022	ALL
**DP**	Categorical Trend ~ *Awareness and Education* + DPSE	4	-18.617	45.609	1.923	0.021	ALL
**DP**	Categorical Trend ~ DPSE + *Action*	4	-18.618	45.609	1.924	0.021	ALL
**DP**	Categorical Trend ~ DPSE + Workforce	4	-18.635	45.644	1.958	0.020	ALL
**DP**	Categorical Trend ~ 1	2	-77.561	159.232	115.547	0.000	ALL
**PS**	Categorical Trend ~ DPSE + *Monitoring and Surveillance* + Workforce	5	-22.292	55.405	0.000	0.068	ALL
**PS**	Categorical Trend ~ DPSE + *Action* + Workforce	5	-22.761	56.344	0.939	0.042	ALL
**PS**	Categorical Trend ~ DPSE + Animal Production + *Action*	5	-22.779	56.380	0.974	0.042	ALL
**PS**	Categorical Trend ~ DPSE	3	-25.194	56.708	1.303	0.035	ALL
**PS**	Categorical Trend ~ DPSE * *General*	5	-23.232	57.286	1.881	0.027	ALL
**PS**	Categorical Trend ~ 1	2	-42.092	88.341	32.936	0.000	ALL
**SE**	Categorical Trend ~ Mean Temperature + *Action* + Workforce	5	-16.861	45.487	0.000	0.098	ALL
**SE**	Categorical Trend ~ *Action* + Workforce + Baseline	5	-17.204	46.172	0.685	0.070	ALL
**SE**	Categorical Trend ~ *General* + Workforce + Baseline	5	-17.208	46.180	0.693	0.070	ALL
**SE**	Categorical Trend ~ *General* + Mean Temperature + Workforce	5	-17.356	46.476	0.989	0.060	ALL
**SE**	Categorical Trend ~ DPSE + *Action* + Workforce	5	-17.457	46.678	1.191	0.054	ALL
**SE**	Categorical Trend ~ 1	2	-25.898	56.120	10.633	0.000	ALL
