# Supplementary material for: Association between national action and trends in antibiotic resistance: an analysis of 73 countries from 2000 to 2023
Source: PLOS Glob Public Health. 2025 Apr 30;5(4):e0004127. doi: 10.1371/journal.pgph.0004127 (PMC12043137; doi:10.1371/journal.pgph.0004127)
Supplement: Attachment — Submitted filename: Response to Reviewers after Ege 241127 - Peter submit 241213.pdf [file pgph.0004127.s036.pdf]

RESPONSE TO REVIEWERS

PGPH-D-24-01686

Association between national policy and trends in antibiotic resistance: an analysis of 73 countries from 2000 to 2023 - PLOS Global Public Health

Please find below our response to all points raised by reviewers. Reviewer comments are highlighted in red and **our responses** in black.

REVIEWER #1

Thank you very much for inviting me to review this interesting work on assessing the national government reporting on AMR action and the change in terms of prevention, mitigation, restoration and adaptation. I must applaud the self-explanatory figures used to explain the process and analysis. However, I have major concern on the clarity of paper been written, presented and the conclusion made.

Thank you for the time and care in reviewing the manuscript. Below we clarify how we have addressed specific comments on clarity, presentation and conclusions.

1) **Abstract.** I could not understand what DPSEEA framework is as it was provided for the first time.

We now mention that indicators relate to drivers, pressures, state and exposure and avoid mentioning DPSEEA in the abstract. This should make the abstract easier to understand.

The study design is not avialable as well as the number of countries included in the analysis.

We now mention the cross-sectional study design in the abstract (cross-sectional regression). We note that the number of countries included in the analysis was already mentioned in the title as well as in the abstract.

How were the countries selected, I guess it was based on the TrACCS. What is the source of government reported policy action and the source of different indicators.

This level of detail is hard to include in the abstract, but it is explained at length in the methods. Now the first section of methods, entitled 'Study design'.

I could not understand the research question until I saw figure 1. Can authors keep it simple. Association of government reported AMR action on the prevention, mitigation, restoration and adaptation. OR Association of government reported AMR action on the health system strengthening, anti-microbial use, anti-biotic resistance and drug resistance.

We have now simplified to something similar to the second sentence.

In the abstract, the second and third sentence is confusing and does not provide good information of the work you are doing, can you please remove it.

We have clarified these sentences so they relate more directly to the research question. They now read:

> The world's governments have agreed both global and national actions to address the challenge of antibiotic resistance. This raises the question of what evidence there is that national action is associated with improved outcomes, including both reduced increases and lowered levels in antibiotic resistance. Answering this question is challenged by variation in data availability and quality as well as disruptive events such as the COVID-19 pandemic. We investigate the association between level of national action and temporal trends in multiple indicators related to health system capacity, antibiotic use (ABU), absolute rates of resistance (ABR) and a drug resistance index (DRI).

2) **Main text**. Introduction- The first paragraph is well written and explains the context. I got lost while reading the second paragraph. In the second para you mention drivers and indicator. Can you please provide the name of indicator. I think there should be a good amount of explanation of four indicators.

We have now added more explanation of the four groups of indicators in the second paragraph. We have also shortened the first paragraph and updated it with current information referring to the recent high-level meeting in 2024. The first two paragraphs now read

Revised:

> Antibiotic resistance (ABR) is a global public health challenge that in 2019 was estimated to contribute to around 1.27 million deaths per year and associated with a total of 4.95 million deaths [1]. In 2015, countries agreed a global action plan (GAP) to address the growing challenge of ABR, which was followed up in 2016 and 2024 with UN high-level meetings, where commitments were made to develop and implement national action plans (NAPs). The high burden of ABR and the increasing focus on national action highlights the importance of assessing the level of action that can be expected to lead to improvements in national conditions. However, such assessment is challenged by multiple factors, including the time it takes from policy adoption to effect of a policy, variation in data availability across countries, and the recent disruption to monitoring of the COVID-19 pandemic [5].
>
> In 2016, the first Global Database for Tracking Antimicrobial Resistance (TrACSS) completed its first assessment of presence and ambition of a standard set of policies in 170 countries. Based on self-reporting, this database gives the first snapshot of what the level of action to tackle antibiotic resistance around the world. Key indicators to consider the effects of policies on span from the upstream drivers that lead to increases in antibiotic use, antibiotic use itself, levels of resistance and the exposure to and impact/burden of resistant infections. Among drivers, lack of health system capacity is a major contributor to inappropriate use. For antibiotic use, the total consumption of antibiotics adds selection pressures for resistance. And the use of broad spectrum and last resort antibiotics are of particular concern. For resistance its relevant to Monitor effects of both common infections as Well as resistance to last resort antibiotics. For exposure and impact, gaps in monitoring mean that good indicators are scarce.

Original

Antibiotic resistance (ABR) is a global health challenge that in 2019 was estimated to contribute to around 1.27 million deaths per year and associated with a total of 4.95 million deaths [1]. In 2015 countries agreed a global action plan (GAP) to address the growing challenge of ABR, which was followed up in 2016 at a UN high-level meeting with commitments to development national action plans (NAPs). In 2024 countries are meeting again at another high-level meeting of the UN to agree the next steps for global and national governance. New goals currently in discussion include the 10-20-30 by 2030 goal to reduce mortality by 10 %, inappropriate human antibiotic use (ABU) by 20 % and inappropriate animal ABU by 30 % by 2030 [2].

Given the severe burden of ABR and the increasing focus on national action, it is important to understand what association there is between action taken so far and improvements in national conditions. The fact that ABR can be limited through multiple points of intervention, including by addressing upstream drivers of ABU complicates assessment [3,4]. Variation in data availability across countries and the recent disruption of the COVID-19 pandemic further adds to these challenges [5]. Instead of associating action taken since the agreement of the GAP with recent trends in ABR, another approach is to see whether countries that were already taking action when the GAP was agreed experienced improved trends in key indicators. Such information will help provide a conservative baseline for the impact of national action on ABR going forwards.

The third paragraph is more of method, it should be in method section. Also, please provide the full form such as DPSEEA, as the acronym is used for the first time.
Please provide your research objective clearly, the research objective as in abstract is not well stated.

The third paragraph is a summary of what the paper tries to do, introducing its aim and overarching method, without going into any detail. Having this at the end of the introduction in our opinion increases the accessibility of the paper for a reader. We have written out the acronyms at first use.

Revised

In this paper we investigate the association of action reported in the TrACSS database with national indicators relating to antibiotic resistance (Fig. 1). Inspired by the Drivers-Pressures-State-Exposure-Effect-Action (DPSEEA) framework, we apply a multi-indicator approach, covering indicators related to drivers (health system), pressures (ABU), state (ABR) and exposure to antibiotic resistant infections in humans (Drug Resistance Index, DRI). Specifically, we construct an action index based on the first TrACSS survey in 2016 and relate to trends in indicators in the preceding 16 years. We apply cross-sectional regression across 73 countries (S1 Table) and model selection to assess the strength of association with linear as well as categorical trends. Finally, we provide assessment of the extent to which countries have increased action from 2016 up until 2023 and assess the prevalence of positively and negatively reinforcing governance responses (Fig 1B).

Original

Here we apply a multi-indicator approach inspired by the DPSEEA family of frameworks to assess the association between action reported in the TrACCS survey in 2016 and preceding national trends in indicators related to drivers (health system), pressures (ABU), state (ABR) and exposure to antibiotic resistant infections in humans (Drug Resistance Index, DRI, Fig 1). We apply cross-sectional regression across 73 countries (S1 Table) and model selection to assess the strength of association with linear as well as categorical trends. Finally, we provide assessment of the extent to which countries have increased action from 2016 up until 2023 and assess the prevalence of virtuous (positively reinforcing) and vicious (negatively reinforcing) governance responses (Fig 1B).

3) **Method.** Can you please use a STROBE checklist to ensure that you do not miss the manuscript reporting. For example, the design is missing. Study setting- The different countries included are missing
Selection criteria
Framework- Figure 1 can be one.

We now use a STROBE checklist for cross sectional studies (v4) for the methods part of the manuscript and changed the text order according to STROBE checklist item orders. We used STROBE checklist titles (Study design, Setting, Variables, Data sources, Bias, Study size, Quantitative variables, and Statistical methods). We excluded participant part since our study doesn't have any participant.
We changed the Figure 1 title from study design to framework. The list of countries included is available in S4 table.

4) **Data analysis**
I am confused why you did an association of action done in 2016 as exposure while the health system strengthen, ABU, AMR as outcome. The reason I can asking this, the Global action plan was endorsed in 2015 and reporting of action was done a year after. How can a any action taken in a year, change the vaccination coverage, sanitation coverage, ABU as these are systematic level changes that requires an optimal years duration. Can you do the validation/reliability assessment instead to see, whether the action reported is true in terms of actions in place. If you keep action as exposure and try to see the temporal trend, it does not fit the standard epidemiological definition.

Our study design and its motivations are explained in detail in study design, the first section of methods. The broad level of policy action is not implemented at one instance, but over a longer set of years.

The survey in 2016 gives an indication of the level of action in a large set of countries. Few countries have likely managed to take significant action since 2015, so the actions reported in 2016 mainly reflect actions that have been implemented over a number of years.

This is why we can justify association the level of action reported in 2016 with trends in the indicators over the preceding 16 years.

Our analysis can be justified even in the case of new action being reported since the adoption of the global action plan. That is because both frontrunner countries and countries taking

action later will be ramping up their ambition on action on antibiotic resistance following the GAP. So, we will still expect the relative level of action in 2016 to be indicative of the general level of action over a number of years.

As we mention analyses after 2016 is challenged by lack of data availability and the disruption of the pandemic.

I really like the indicators selection, can you please make a table and show within each drivers what were the indicators (denominator, numerator) and source of data.

The indicators were given in S2 Table, now expanded into S1-S3 Table, we have updated the table so that it includes information about denominator and nominator and a column for the source.

We have updated the text so it now explicitly mentions that “Indicator selection and tiers and data sources are mentioned in detail in S1-S3 Table.”.

5) **Result-** The result are well presented especially with the graphs.

Thank you for your comment.

6) **Discussion** Can you please method a section on methodological consideration, which is missing.

We have inserted a section on limitations at the bottom of the discussion before conclusions. It reads:

Our study has several limitations that should be taken into account when interpreting the results. First, our study is limited by the data availability in countries, which vary by indicator group and is generally biased towards high-income countries. Care should be taken when interpreting the results to not transfer them outside of their context. For specific income-settings, results relating to the specific high-income model and the model for low- and middle-income countries should be used. The level of action is self-reported by countries and does not necessarily reflect the actual level of policies implemented in the country. Further, we cannot account for how long policies have been implemented. For example, it is reasonable to assume that countries with higher policy ambition levels have been frontrunners in the field for some time, policies therefore have been in place longer, and potentially showing a larger effect compared to countries who have recently implemented policies. Our data is limited to antibiotic indicators in the human sector and does not necessarily say anything about the level of action needed for reducing adversity or improving trends in crop or animal health systems.

REVIEWER #2:

I must commend the ingenuity in applying regression statistical analysis to the DPSEEA framework to assess the association between national policies and trends in antibiotic resistance for 23 years across 73 countries. This approach not only adds a novel dimension to antibiotic resistance research but also provides valuable insights that could guide future interventions.

Thank you.

While your manuscript provides a comprehensive overview of the indicators and data sources, I noticed that specific datasets or versions are not always mentioned (e.g., Lines 125 and 160). Perhaps, you could state, 'The data used for antibiotic consumption trends were sourced from the QuintilesIMS MIDAS database, version X.Y (DOI: xxxx).'
Explicitly providing DOIs, URLs, and any conditions for data access would greatly enhance the transparency and reproducibility of your study, in line with PLOS's data policy. Including these details will not only strengthen your manuscript but also make it easier for other researchers to build on your work.

We included a citation according to your suggestion. Since there is no DOI or URL or version for the IQVIA data (it is a proprietary company that provides the data at exorbitant fees and the name of company has changed to IQVIA). But it is now all publicly available on the ResistanceMap website (https://resistancemap.onehealthtrust.org/). We used relevant citation for Antibiotic use and Antibiotic Resistance data as suggested at ResistanceMap website (Citation number 10 and 13) such as in the example below.

We use antibiotic consumption data for humans obtained from the IQVIA database [10] for the years 2000 to 2015.

We switched S3 and S4 table order and updated the S3 Table description as follows: "Questions used and their sectors included for the calculation of governance syndrome for the years between 2016-2022 according to TrACSS (1.1) 2016-2017 version. All surveys are available online (https://amrcountryprogress.org/#/download-responses)." Furthermore, we updated line 160 as with the version number: "Country selection. Countries included in the various analyses were filtered based on two criteria. First, answering the TrACSS (1.1) survey in 2016-17 (S3 Table)."

7) Moreover, I recommend explicitly referencing the datasets where not already done (line 125 and 160) or including all of them in supplementary materials. This additional detail will further solidify your manuscript's contribution to the field and ensure that it is a model of transparency in antibiotic resistance research.

We updated the text to "Indicator selection and tiers and data sources are mentioned in detail in S1-S3 Tables." And put the data sources in S1-S3 Table for each indicator..

/ END OF DOCUMENT
